# Memory in the digital age

**DOI:** 10.12688/openreseurope.16228.1

**Published:** 2023-08-04

**Authors:** Silvana Mandolessi

**Affiliations:** 1 Cultural Studies, KU Leuven Association, Leuven, Flanders, 3000, Belgium

**Keywords:** Digital memory, collective memory, memory studies, forgetting, archive

## Abstract

This article explores the configuration of collective memory under the impact of the digital turn. In recent debates, there has been a marked tendency to interpret ‘digital memory’ as a new type of memory, which is radically different from the traditional conceptualization. Even leading authors in the field claim that the digital revolution implies the end of collective memory. However, I argue that despite the transformations that memory undergoes in the digital age, these changes do not imply a new ontology of memory but rather a materialization of the theoretical claims made by Memory Studies since the field's inception.

To support this hypothesis, I analyze digital memory in relation to three topics: first, I focus on the problematic definition of collective memory to demonstrate how the digital realm allows us to rethink the social nature of memory through a different concept of the social. By contrasting Halbwachs' notion of the social, which forms the basis of memory studies, with the alternative proposal of Gabriel Tarde, I argue that the latter enables us to refine the concept of the ‘collective’ that we have inherited from the founding figure of memory studies. Second, I delve into the new ontology of the digital archive showing how it materializes one of the defining features of collective memory: its mobile, dynamic, and procedural nature. Lastly, I address the inversion of the dialectic between memory and forgetting to highlight the specificity of these practices in the digital environment. I demonstrate how these changes effectively implement, surpassing older technologies, the concept of collective memory as a distributed and dynamic technological process that shapes our shared representations of the past.

## Plain language summary

In everyday language, memory is conceived of as an individual faculty; however, remembering is an eminently social act. Societies elaborate representations of the past that are fundamental to the constitution of their identity. Following this, the field of memory studies has explored the way collective memory, the relationship that a social group establishes with 'its' past, is socially constructed and circulates, as well as its multiple effects on our experience of the present. One of the fundamental insights of memory studies is that memory is always mediated. Different media shape memory in different ways, providing memory with specific affordances and constraints. If the advent of writing in early civilisation radically altered the constitutions of memory cultures, and the invention of print marked a new shift putting memory in circulation in an unprecedented way, the onset and spread of digital media signals the more recent revolution in collective memory and mnemonic communities. This article explores the configuration of collective memory under the impact of the digital turn. Recent debates suggest that ‘digital memory’ is a completely new form of memory that is fundamentally different from traditional memory. I argue that while memory has certainly transformed in the digital age, these changes don’t create a new type of memory but materialize the theoretical claims made by memory studies from its inception. To support this argument, I examine digital memory in three key aspects. First, I discuss the definition of collective memory and how the digital realm allows us to reconsider the social nature of memory. Second, I explore the new nature of digital archives and how they embody the essential characteristics of collective memory: mobility, dynamism, and procedural nature. Lastly, I address the inversion of the dialectic between memory and forgetting to highlight the specificity of these practices in the digital environment.

## Introduction

The impact of the digital turn on our culture is undeniable. It has revolutionized the way we consume and create content, breaking down geographical barriers, and empowering individuals to participate in cultural expression on a global scale. With the rise of social media, digital media has also transformed how we communicate and interact with each other, changing the way we form communities and share ideas. One of the major questions that are systematically asked in the debates on the digital is the scope and stakes of this impact. Is the digital turn a real break with previous cultural frameworks? Or, on the contrary, it is better to think the digital as a remediation of the older media? In this article, I aim to examine the impact of the digital turn on ‘collective memory’ *.* Following Schwartz, collective memory can be defined as “the distribution throughout society of what individuals know, believe, and feel about the past, how they judge the past morally, how closely they identify with it, and how much they are inspired by it as a model for their conduct and identity” ( Schwartz, 2016, 10)

Since what has been called the ‘memory boom’ in the 80s, collective memory has been an object of continuous academic inquiry as well as a constant presence in public debate. Fueled by various factors —including the exceptional scope of violence and wars of the 20th century, changes in media technology, the end of the Cold War, and developments within academia ( Erll, 2011a, 4–5)— the memory boom led to a detailed investigation of the role of memory in the formation of collective identities, its political function, its stability and variability over time and space, its fidelity or distortion of historical events, its ‘insufficiency’ or ‘excess’ ( Connerton, 1989; De Cesari & Rigney, 2014; Levy & Sznaider, 2006; Olick *et al.*, 2011a; Radstone & Schwarz, 2010; Rothberg, 2009; Tota & Hagen, 2016).

In the last 30 years, memory studies not only became institutionalized, as evidenced by the existence of specialized journals and editorial collections, but they also, in some way, became standardized —or at least a part of them— around an approach to the representation of the past that ended up being repetitive and sterile. Assessing the current state of the field and the future of memory studies, Astrid Erll stated,

“With the methodology at hand, memory studies will easily be able to keep generations of scholars busy, charting the mnemonic practices of all ages and places. However, the question arises whether ‘memory’ is thus turning into a mere ‘stencil’, and memory studies into an additive project: we add yet another site of memory, we address yet another historical injustice. While such memory work is for many historical, political and ethical reasons an important activity, memory research finds itself faced with the decisive question of how it envisages its future” ( Erll, 2011b, 4)

Erll's critique of the need to update the conceptual and methodological framework of memory studies is even more evident in the contemporary scenario. Today, it is impossible to understand the shaping and transmission of collective memory without addressing the changes brought about by the digital turn. However, although there is consensus on acknowledging the impact of electronic media —something that would be futile to deny— the extent of these changes is a subject of debate. Is it an evolution or a revolution? Are we witnessing a radical break from pre-digital forms or a cultural continuity where mnemonic practices do not undergo substantial modification?

In the recent debate, the prevailing idea is that digital memory represents a radical change, giving rise to a new form of memory referred to as “algorithmic memory,” “connective memory,” “new memory,” or “the memory of the multitude” ( Blom, 2017; Garde-Hansen *et al.*, 2009; Makhortykh, 2021; Hoskins, 2009; Hoskins, 2011; Hoskins, 2018b). According to these perspectives, digital memory not only implies a different ontology but, in more radical proposals, even entails the “end of collective memory” ( Hoskins, 2018a). Are we indeed facing the end of collective memory? Should we abandon the conceptual framework and insights gained along the way, closing the debates that have occupied us by deeming them irrelevant in the contemporary setting? I would claim the opposite. Digital memory is not a new form of memory and, far from representing the end of collective memory, it materializes and puts into practice the characteristics with which we have defined collective memory since the inception of the field. In fact, we could argue that what we have always advocated about collective memory finds its true realization in the digital era.

But before presenting the arguments that support my hypothesis, I would like to clarify what I understand by digital memory and in what sense I affirm that this memory is not new.

To clarify the meaning of digital memory, it is helpful to recall the distinction between ‘medium theory’ and ‘media theory’. ‘Medium theory’ focuses on the medium as a material communication channel, while ‘media theory’ emphasizes the notion of medium as a social practice. The different approaches of both theories correspond to two different ways of understanding a medium. As Baetens asserts, "In the narrow sense of the word, a medium is a channel. In the broad sense of the word, it is a culture" ( Baetens *et al.*, 2020, 81). When understood as a channel, what matters in a medium are its specific traits that define it as such, differentiating it from other media. In the field of memory, an example would be the theories that attribute specific characteristics to photography as a mnemonic medium, such as its ‘indexicality’ or Barthes's ‘that-has-been’ element in *Camera Lucida.* In contrast to the definition that confines a medium to a channel, a medium understood as culture examines how the emergence of a new medium modifies the overall media ecology, giving rise to new social practices or substantially modifying existing ones.

The prevailing approach in most reflections on digital memory is framed within the understanding of a medium as a culture rather than merely a communication channel. This choice is based, first and foremost, on the difficulty of confining ‘the digital’ to a specific medium since the digital remediates—using the term popularized by Bolter and Grusin in their seminal study *Remediation: Understanding New Media* ( 1999)—previous media, ranging from television and photography to literature and cinema. This does not imply disregarding the importance of a medium approach that adheres to the thesis of the digital as the epitome of a post-media condition. For several reasons a "medium approach" remains essential (see Baetens *et al.*, 2020, 101–2). However, the debate about digital memory is not limited to digitally born mnemonic objects —for example, an online memorial, databases of survivors’ testimonies, or a project like ‘Anne Frank. A Cold Case Diary’ —but it explores how collective memory is reconfigured under the impact of digital media, or, to put it differently, how digital culture gives rise to a new mnemonic culture.

Regarding the second point: when I state that digital memory is not new, I do not mean to say that memory has not undergone changes under the impact of the digital realm—which would be absurd to claim. On the contrary, the changes are profound and they affect both the actors involved, as well as the objects and practices. What I propose is that these changes, instead of constituting a new memory, actually materialize or implement the essential characteristics that define collective memory. What digital memory offers us is a memory that can now align more perfectly with its own definition, bridging the gaps between theory and practice. It also allows us to reconsider problematic aspects in theory under a new light.

In what follows, I will examine digital memory in relation to three topics. First, I will focus on the problematic definition of collective memory to demonstrate how the digital realm allows us to rethink the social nature of memory through a different concept of the social. By contrasting Halbwachs' notion of the social, which forms the basis of memory studies, with the alternative proposal of Gabriel Tarde, I argue that the latter enables us to refine the concept of the ‘collective’ that we have inherited from the founding figure of memory studies. Second, I will delve into the changes experienced in the archive —an essential concept in the debate on digital memory, as evidenced by seminal works such as *Digital Memory and the Archive* ( 2013) by Wolfgang Ernst, *Rogue Archives: Digital Cultural Memory and Media Fandom* ( 2016) by Abigal De Kosnik, or the edited volume *Memory in Motion: Archives, Technology, and the Social* ( 2017) by Blom, Lundemo, and Røssaak. The new ontology of the digital archive materializes one of the defining features of collective memory: its mobile, dynamic, and procedural nature. Lastly, I will address the inversion of the dialectic between memory and forgetting to highlight the specificity of these practices in the digital environment. I will demonstrate how these changes effectively implement, surpassing older technologies, the concept of collective memory as a distributed and dynamic technological process that shapes our shared representations of the past. 

## Rethinking the social

What do we mean when we use the term collective memory? How do we conceptualize the collective dimension as distinct from individual memory? This has been —and continues to be— a highly debated issue in memory studies. Critiques point out that despite extensive interdisciplinary research in recent decades, the concept of collective memory remains problematic due to its vagueness, imprecision, and limited effectiveness in describing an elusive phenomenon ( Kansteiner, 2002; Olick & Robbins, 1998; Schwartz, 2016; Sturken, 2008; Wertsch & Roediger, 2008).

How do we empirically observe what we call collective memory? Following the fundamental assumption that memory is always mediated ( Garde-Hansen, 2011; Neiger, 2020; Neiger *et al.*, 2011; Van Dijck, 2007; Van House & Churchill, 2008) and that direct access to shared representations of the past is impossible, collective memory is observed through its material inscriptions, in the expressions that construct, preserve, and transmit our knowledge and emotional connection to the past —or to significant events of that past. The mnemonic media studied include literature, film, photography, music, theater, memorials, statues, but also mediums such as landscape, orality, and the body. The problem lies in the fact that the study of mnemonic media only seems to provide us with fragmentary and incomplete access to collective memory, understood as a representation shared by the social group. To what extent do films like *Schindler's List, Shoah*, or the Berlin Holocaust Memorial reflect the collective memory of the Holocaust? Do all members of the social group share it? What different images or narratives are excluded from those representations? Although it is theoretically emphasized that collective memory is a fluid and dynamic constellation, an arena of struggle where different narratives about the past collide, the term collective memory still carries the imaginary of a homogeneous and essentially shared entity among the members of the social group. The study of its inscriptions, therefore, seems to be constantly torn between the fragmentation or dispersion of partial representations and the desire to capture an elusive totality that exists above or beyond individuals. This problematic articulation between the individual and the collective has its origins in the father of memory studies, Maurice Halbwachs.

In 1925, Maurice Halbwachs published *Social Frameworks of Memory* ( *Les cadres sociaux de la mémoire*), a book in which he advanced the concept of ‘collective memory’. While Halbwachs was not the only one to address the social nature of memory at that time —Aby Warburg is often cited as representing an alternative genealogy in the field— the current concept of collective memory derives from his reflections ^1^.

Halbwachs's exploration of memory integrated perspectives from two influential figures in late nineteenth-century France, philosopher Henri Bergson and sociologist Emile Durkheim ( Olick *et al.*, 2011a, 30–36). Bergson conducted a radical philosophical analysis of the experience of time, emphasizing the central role of memory within it. He challenged the notion of memory as a passive storage mechanism and instead characterized remembering as an active process. Bergson's exploration of memory brought to Halbwachs's attention the distinction between objective (often transcendental) and subjective perceptions of the past. Against the uniform time of the clock, individual memory was essentially variable and dynamic, vividly capturing short periods while leaving longer periods only vaguely outlined.

Durkheim agreed with Bergson in rejecting transcendentalist explanations of time but, unlike Bergson, Durkheim attributed the variability of perceptual categories not to subjective experiences but to differences among various forms of social organization. According to Durkheim, groups share ‘collective representations’ —narratives, symbols, meanings— that constitute the group but not need to be shared by all members of the group. This is the reason Durkheimian approaches has been often criticized “for being radically anti-individualist, conceptualizing society in disembodied terms, as an entity existing in and of itself, over and above the individuals who comprise it” ( Olick *et al.*, 2011a, 30). Another important characteristic, as Olick, Vinitzky-Seroussi, and Levy highlight, is “the tendency to assume, without justification, that these societies constituted by ‘collective representations’ are homogenous entities. Consequently, a Durkheimian approach to collective memory can lead us to attribute a single collective memory or a set of memories to entire, well-defined societies” (33). As J. Olick, Vinitzky-Seroussi, and Levy summarize, “by connecting cognitive order (time perception) with social order (division of labor), Durkheim thus provided for Halbwachs a sociological framework for studying the variability of memory raised by Bergson” ( Olick *et al.*, 2011a, 31).

It is this conception of collective memory as an abstract, disembodied, and homogeneous entity that has been criticized for not providing an adequate framework to capture the social nature of remembrance. In the current digital environment, this inadequacy becomes even more apparent.

 Following a Durkheimian approach, the term ‘collective’, in the classical theory of Halbwachs, refers to social groups with a dynamic but stable identity. Even if an individual can be part of several groups, they appear as clear-cut formations, unified by a shared identity, a set of shared values, and attached to a territory. Today, on the contrary, the social —as in social media— is the product of a social engineering driven by the entanglement of algorithmic forces, shared digital values, and participatory practices. The meaning of ‘social’ hence encompasses both (human) connectedness and (automated) connectivity, since social media are inevitably automated systems that engineer and manipulate connections, by coding relationships between people, things, and ideas into algorithms ( Van Dijck, 2013, 12). As Latour (2005) puts it, the social is not a thing or domain, not an explicative category but precisely what needs explaining. This becomes even more acute in the digital era, in which the social is not a fact but a doing, performed through digital connections ( Bucher, 2015). As Blom wonders about the suitability of the concept of collective memory in the digital age,

“But what if the material frameworks of memory seem to lack the type of stability and durability that confer identity on things? What is a society’s self-image if this image may be the object of instantaneous erasure, dispersal through multiple relays or information overflow, or transmutation through dynamic feedback circuits? What is society if its memory images are perhaps not even representations?” ( Blom, 2017, 14).

The question is: what conception of the social would allow us to understand the collective nature of memory in the digital era? Gabriel Tarde's theory may provide us with an answer. ^2^ It can constitute an alternative for rethinking the social ontology inherited from Durkheim, which forms the basis of Halbwachs’ conception. It is about conceptualizing the collective nature of the binomial ‘collective memory’ in a way that reflects both the heterogeneity of multiple individuals and what allows us to speak of a shared memory —that is, something that goes beyond the individual but does not negate it.

The first relevant point in Tarde’s view is a conception of the social that is not limited to human relationships. Not only are human beings social, but social behavior is inherent in all phenomena in the universe, from atoms, nations, cells, and bacteria to plants and animals. As Tarde remarks, “every thing is a society and every phenomenon a social fact” ( Tarde, 1999, 58).

According to Tarde, the ‘social’ consists of the capacity for association or aggregation that all elements possess, which is evident even in the smallest unit —the monad. Although Tarde's vision has been accused of methodological individualism, in truth, the individual does not exist in his view because the individual element is already a monad, that is, an entity composed of a multiplicity of aggregated elements. Social phenomena emerge from the interactions and imitations between monads. Monads are interconnected through a complex network of social relations and influence each other through processes of imitation. Tarde believed that imitation was a crucial mechanism driving social change and the diffusion of ideas, behaviors, and innovations within society.

The events of association between monads never crystallize into a structure. The whole is always less complex than the sum of its parts, since the heterogeneity of the elements that constitute the monads can ally with other ‘wholes’. As Tarde puts it:

“The internal uprisings which finally destroy all of these great, regular mechanisms —the social mechanism, the vital mechanism, the stellar mechanism, the molecular mechanism— are all due to a similar condition: their constitutive elements, soldiers of these various regiments, temporary embodiment of their laws, only ever belong to the world they constitute by one facet of their being, while by other facets they escape it. This world would not exist without them; they however would still be something without it. The attributes which each element owes to its incorporation within its regiment is not the whole of its nature. It has other tendencies, other instincts, which come from other regimentations ...” (1999: 39) ( Candea, 2010, 9).

The task of sociology would then be to trace the path of monads converging into aggregates without losing their individuality. To be able to grasp the trajectory of associative events when they temporarily stabilize and return to individual variations. In other words, to make visible the associations that produce ‘the collective’ without subsuming the collective under ‘structure’, ‘social law’, or ‘representation’. This is precisely what digital technologies allow us to do today and what, according to Latour, would demonstrate that Tarde was right. Latour suggests that the theory of ‘society’ elaborated by Durkheim was “an artifact of rudimentary statistics,” ( Latour, 2010, 157) as they had to obliterate individual variations to obtain the similarities that constitute a pattern.

On the contrary, today “we are able to physically (well, virtually) navigate on our screens from the individual data points to the aggregates and back. In other words, the aggregate has lost the privilege it maintained for one century. Through the ease with which we can navigate a datascape, we manage to interrupt the transubstantiation of the aggregate into a law, a structure, a model, and complicate the way through which one monad may come to summarize the ‘whole’ ( Latour, 2010, 158). As Latour contends, “We can now have our cake (the aggregates) and eat it too (the individual contributors)” (158).

With the rudimentary statistics of the past it made sense to treat data about social connections by defining two levels: one for the element, the other for the aggregates. “But once we have the experience of following individuals through their connections (which is often the case with profiles) it might be more rewarding to begin navigating datasets without making the distinction between the level of individual component and that of aggregated structure” ( Latour *et al.,* 2012, 590) In this sense, Latour argues, “it becomes possible to give some credibility to Tarde’s strange notion of ‘monads’” ( Latour *et al.,* 2012).

If Halbwachs' theory was the result of addressing the variability of memory raised by Bergson through Durkheim’s sociological lens, it would be necessary to reconsider the variability of Bergson, but through Tarde's perspective. Tarde’s approach provides a framework for conceptualizing the social as the always provisional and dynamic outcome of associative events. Collective memory is not a disembodied and homogeneous representation that exists in itself, above and beyond the individuals who make up society. Instead, the collective is constituted as an aggregated entity but internally heterogeneous, with its stability always being provisional. As Latour suggests, digital technologies allow us today to put Tarde's theory into practice by observing the process through which individual elements come together to form a collective that preserves the inherent heterogeneity and dynamism of its individual components.

In summary, Tarde's conception of the social allows us to retune the concept of collective memory in three ways. Firstly, it enables us to replace the notion of collective memory as an abstract, disembodied, and homogeneous entity that exists in and of itself, over and above the individuals who comprise it, with a series of heterogeneous and dynamic performanes of the past that result from associative events among monads. These associative events are not only between individuals forming aggregates but also the associative events individuals establish between the past and the present.

Secondly, Tarde’s conception allows for the integration of theories that have challenged traditional notions of collective memory by highlighting the interconnected and mutually influencing nature of memories across different historical events and communities, such as Michael Rothberg's influential “multidirectional memory” ( 2009). According to Rothberg, collective memory is not confined to specific groups or events but is a complex network of memories that intersect, overlap, and interact with one another. Rather than seeing memory as a fixed and exclusive narrative, Rothberg argues that memories are dynamic, evolving, and fluid. Tarde’s idea that the whole is always less complex than the sum of its parts —since the heterogeneity of the elements that constitute the monads can ally with other wholes— provides a foundation for understanding how memories of different groups intersect and interact with one another. 

Finally, by not restricting the social to human actors, Tarde's theory allows us to explore collective memory as a process encompassing both (human) connectedness and (automated) connectivity. The role of algorithms in encoding the relationships between people, things, and ideas is essential for understanding how shared representations of the past are formed and transmitted in the contemporary landscape. Algorithms play a crucial role in shaping the way information is curated, filtered, and presented to individuals within digital platforms and networks. They influence what content is prioritized, recommended, and made visible to users, thereby shaping their understanding and perception of historical events, cultural narratives, and collective memory. Understanding the role of algorithms in the encoding of relationships and the formation of shared representations of the past is crucial for critically analyzing and navigating the contemporary digital landscape. It prompts us to consider the potential biases, limitations, and power dynamics inherent in algorithmic systems and their impact on collective memory. Based on a conception of collective memory indebted to Durkheim's paradigm, this exploration is simply impossible.

## The archive in motion

In theorizations of memory, the archive has arguably been the most powerful and enduring metaphor. Well-known analogies of memory, such as Plato’s wax seal or table, the loci of classical art or Freud’s mystic writing-pad, rely on the “imprints-on-a-substrate paradigm”, an imagery that conceives memory as a content preserved in a specific place —whether it's the brain, the library, or the screen ( Burton, 2008, 322). The trait that defines the archive in these metaphors is stability. The archive extracts something from the flow of time and safeguard it from decay, preserving it 'intact' for the future.

Clear examples of this can be found in well-known theories on collective memory, such as the opposition between ‘canon’ and ‘archive’ proposed by Aleida Assmann. For Assmann, “the institution of the archive is part of cultural memory in the passive dimension of preservation” ( Assmann, 2008, 103). The knowledge saved in the archive is inert, “it is stored and potentially available, but it is not interpreted” (103). While the archive refers to the passive dimension of cultural memory, the canon “stand for the active working of memory of a society that defines and supports the cultural identity of a group” ( Assmann, 2008, 106). In a similar vein, Diana Taylor opposes two epistemological systems, coded in the dichotomy ‘archive/repertoire’. ‘Archival’ memory exists as documents, maps, literary texts, letters, archaeological remains, bones, videos, films, and all those items supposedly resistant to change. The repertoire, on the other hand, enacts embodied memory and encompasses mnemonic practices such as performances, gestures, orality, movement, dance, or singing ( Taylor, 2003).

In the digital age the static archive is replaced by an “archive in motion” ( Røssaak, 2010). The archive, conceived as the guarantor of the stability of collective memory, undergoes an ontological change characterized by dynamism and mobility. This dynamism is evident in different domains and levels, involving diverse topics such as techno-mathematical operations governing data transfer, access to previously unavailable materials, and the emergence of new archival practices carried out by amateur communities.

The complexity of the debate surrounding the relationship between the archive and digital technologies lies in the fact that, as Taylor argues, archive simultaneously refers to a place, an object, and a practice.

“An archive is simultaneously an authorized place (the physical or digital site housing collections), a thing/object (or collection of things —the historical records and unique or representative objects marked for inclusion), and a practice (the logic of selection, organization, access, and preservation over time that deems certain objects “archivable”). Place, thing, and practice function in a mutually sustaining way” ( Taylor, 2012).

The changes experienced by the archive in the digital era are observed in the three domains.

Firstly, the archive as thing/object. What is stored in an archive can be any object, ranging from the text of a law, a literary work, a painting, the recording of a musical score, to an Aztec’s mask. All these tangible and material things are transformed into the logic of zeroes and ones. Although digital technologies may speak the language of storage and containment, as Blom argues, in digital media “nothing is stored but code: the mere potential for generating an image of a certain material composite again and again by means of numerical constellations ( Blom, 2017, 12). Blom emphasizes mobility, asserting that “once the archive is based on networked data circulation, its emphatic form dissolves into the coding and protocol layer, into electronic circuits or data flow” (13).

Objects in the archive have, of course, always circulated. In fact, the purpose of an archive is not only to preserve documents but also to make them available in a potential future. The difference is that preservation now relies on inherent dynamism, as the conservation of the object in the logic of zeros and bytes involves its translation into protocols and codes that are then updated again when we request the image or text to read it through the interface. The dynamism of digital objects lies in these algorithmic operations formed by constant regenerations, updates and affordances. Archives are “dynamic living systems, constantly transformed and updated” ( Røssaak, 2017, 202–4). Or as Blom puts it: “The conflation of memory with storage is, in other words, undermined by a technical emphasis on dynamic processes of memorizing. To the extent that computer memory exists, it is essentially activity; virtual as well as actual, and its images are electronic events ( Blom, 2017, 12).

Secondly, the archive as place. The spatial arrangement of an archive within a public building has been replaced by the ‘placelessness’ of documents distributed along multiple sites. Digital memories are often perceived as 'placeless', meaning that one notable change brought about by digital media is the diminished connection to a specific location. Under this perspective, memories in the pre-digital era were strongly tied to physical spaces, which played a crucial role in their interpretation. However, in the present era, this connection fades away, leading to memories that are fluid, detached, fragmented, and omnipresent, constantly shifting without precise spatial references. The loss of a meaningful bond with place connotes, in this imaginary, a loss of meaning, given the centrality that the category of place plays in the shaping, preservation, and transmission of collective memory. The idea of placelessness is linked to the changing traits of the archive and the database as the privileged cultural forms of the digital era. The notion of the traditional archive as static, selective, organised, and restricted to a particular place, has been replaced by a dynamic, non-selective, and multimedia online archive, whose logic is fluidity and ubiquity, and which is always ‘in becoming’ ( Mandolessi, 2021)

However, this imaginary has been questioned by highlighting how digital memory practices actively engage in place-making, rather than succumbing to the logic of hyper-connectivity and all the attributes upon which it is predicated ( Mandolessi, 2021). Rather than deterritorizaling memories, digital archives become key tools for new engagements with place (for different approaches on the issue of digital memory and place see the Special Issue “Locating “Placeless” Memories: The Role of Place in Digital Constructions of Memory and Identity”, edited by Huw Halstead, Memory Studies 14.3 (2021).

Thirdly, the archive as a practice. The practice of archiving involves the selection and organization of objects that will be stored in the archive. The authority of experts is crucial, as the decision to archive certain documents and discard others grants them a legitimization that they do not possess prior to their entry into the archive. In this sense, archivization is performative, its operations of selection and organization, as Derrida claims “produces as much as it records the event” ( Derrida, 1996, 17). The importance of selection is emphasized by Ketelaar, who coined the term ‘archivalization’ to denote a phase prior to the archiving process, meaning “the conscious or unconscious choice (determined by social and cultural factors) to consider something worth archiving. Archivalization precedes archiving” ( Ketelaar, 2001, 133)

The digital has radically altered the phase of ‘archivalization’ in at least two senses. Digital media are inherently archival, meaning that even if we do not consciously decide to archive specific content, it will be automatically stored. For example, our tweets or posts on social media platforms like Twitter or Instagram, the locations we visit through geo-tagging, or our selections on online shopping platforms like Amazon. There are no experts behind the scenes determining which content is worthy of archiving; instead, algorithms are designed to collect and process data. This automation is closely tied to the idea of the Internet as a ‘perfect memory machine’ capable of recording and preserving everything. The persistence of data challenges our right or desire to forget, to leave certain things in the past (which I will address in the next section). The lack of selection in automated storage seems to complicate matters rather than offering a solution to the scarcity of memory.

However, changes in archival practices not only involve algorithms recording information with or without our consent but also encompass alternative selection criteria that go beyond traditional notions of expertise. In the digital era, archives can be constructed by anyone who believes that certain materials are worth archiving.

In her book *Rogue Archives: Digital Cultural Memory and Media Fandom* ( 2016), De Kosnik extensively explores the work of technovolunteer archivists. These ‘rogue archives’ are established and maintained by fans with the aim of preserving and sharing cultural artifacts that are often neglected, banned, or considered outside the mainstream cultural canon. The efforts of these technovolunteers play a crucial role in curating and safeguarding the cultural memory of marginalized groups, rather than relying solely on the assumed archival nature of the internet. These archives possess a democratizing potential that challenges the criteria upheld by official institutions regarding what should be preserved or not. This expansion of value in determining preservation challenges the established norms and opens up possibilities for a more inclusive approach to archiving. Moreover, rogue archives not only enable the preservation of materials that would otherwise be lost but also fundamentally transform the mnemonic function of the archive. According to De Kosnik, the archive, which was traditionally seen as a record of cultural production, now becomes a source of cultural production itself. She introduces the term “rogue memory” to signify this shift from preservation to creativity.

Returning to the question posed in the introduction: In what way does the new ontology of the archive materialize the concept of collective memory?

Collective memory is defined as a practice, an active process that interprets the past in light of the present. However, the metaphor of the archive pointed to a static content, those objects, traces, and remnants of the past that could potentially be activated but also remain inert. This potentiality gave the archive a liminal or ambiguous status. If we define memory as an active process, in what sense can we speak of the archive as a ‘passive dimension of memory’? The new ontology of the digital archive, an archive in motion, brings together potentiality and actualization. In the digital era, practices are inscribed in the archive, which no longer functions as a mere passive container or collection of traces of the past but as a dynamic entity. The archive becomes the site in which the traces of the past are continuously interpreted, updated, and collectively reappropriated in an ongoing process.

## The inversion of the dialectics between memory and forgetting

In principle, collective memory refers to any shared representation of the past by a social group. However, the meaning of memory that dominates the field of memory studies has a more restricted character. It is a memory of violence, atrocity, and human right abuses ( Huyssen, 2011; Levy & Sznaider, 2006). This focus on memory centered on violence is deeply linked to the emergence and globalization of a powerful discourse on human rights, which is materialized in the adoption of the Universal Declaration of Human Rights in 1948. In this foundational text, memory holds a central place as an instrument to promote the defense of human rights, a role summarized in the ‘duty to remember.’ There are two underlying assumptions regarding the ethical and moral responsibility to remember that form the core of the relationship between memory and human rights: The first assumption is that acknowledging human rights abuses and honoring victims through memory represents the ethically correct and necessary response to violence. The second assumption further intertwines memory with human rights: the remembrance of past violence is considered one of the most effective measures to prevent future violence ( Sodaro, 2018). The link between memory and human rights explains the positive role assigned to the act of remembering and the corresponding stigmatization of forgetting. This also helps to explain why, in comparison to the extensive research on the various ways in which societies remember, there is limited focus on how societies forget. In general terms, forgetting has been viewed as something to be combated rather than as an object that requires scrutiny. One notable exception is Paul Connerton's *Seven Types of Forgetting* ( 2008) and *How Societies Forget* ( 2009). Connerton does not stigmatize forgetting. On the contrary, he demonstrates the variety and complexity of the mechanisms involved in the act of forgetting and how forgetting can be necessary, for various reasons and in different contexts, for political and social life.

This lack of interest in forgetting as an object of study —its forms, its actors, its means, and its value— is currently being reversed, becoming central in the debate on digital memory. The value attributed to forgetting is also being inverted. Beyond memory associated with human rights, where the ‘duty to remember’ prevails, the question about the function and benefits of forgetting is being brought back into the spotlight. In the digital age, the dialectic between memory and forgetting has been reversed As Mayer-Schönberger claims “Quite obviously, remembering has become the norm, and forgetting the exception” ^3^ ( Mayer-Schönberger, 2009, 52). We need to forget. To forget what? To forget how? And why? The debate can be structured around these questions.

In the debate about memory in the digital era, the essential function of forgetting as an individual and social mechanism is affirmed. The importance of forgetting has been even ratified by a legal framework known as ‘The Right to be Forgotten’ ^4^. ‘The Right to be Forgotten’ is a legal concept that refers to an individual's right to request the removal or deletion of personal information from online platforms and search engine results. It recognizes the importance of privacy and allows individuals to have control over their personal data and its availability on the internet.

Despite the existence of a legal framework that guarantees the right to be forgotten, the question of whether it is possible to forget —or be forgotten— in a digital environment remains. Is it true that forgetting becomes impossible in the digital era? Or, on the contrary, is it true that collective memory comes to an end? Perhaps, these bold claims about the impossibility to forget or the impossibility to remember in a digital era stem from a conception of memory and forgetting that remains confined to the framework of an analog or pre-digital culture. It would be more appropriate to examine the specific ways in which we forget and remember in the current media ecology. Following this line of thought, I would like to briefly discuss two examples of alternative forms of (digital) memory and forgetting explored by Elena Esposito and Serge Bouchardon.

## Re-inventing Forgetting

In ‘Algorithmic Memory and The Right to be Forgotten’ ( 2022), Elena Esposito discusses a judgment that the European Court of Justice issued in 2014 in favor of the plaintiff on case C-131/12 about the ‘right to be forgotten’. The European Court held Google accountable for this ‘excess of memory.’ On the other hand, Google claimed that it could not be held responsible, arguing that the processing of data is carried out by the search engine, and the company does not exercise control over that data. However, according to the European Court, although Google may not be directly responsible for data processing, the search engine’s activity makes that data accessible to users. Esposito remarks that this raises the question of what conception of social memory and forgetting is implied in the ruling:

“Is memory the ability to store information in an archive, even if it is inaccessible? Or does it depend on the ability to find the information when you need it? Is computer memory storage or remembering? Ascribing to Google the management of the right to oblivion implies a clear choice: data are considered forgotten if they are made difficult to find, while social memory should be preserved by the storage of data in the pages of newspapers and in other archives” ( Esposito, 2022, 67–68).

Esposito acknowledges the difficulty of requesting certain information to be erased, among other reasons, because it draws attention to the deleted content, achieving the opposite effect of what is desired. How, then, to deal with forgetting on the web? Esposito suggests that forgetting should follow the logic of algorithmic memory by implementing a procedure that involves multiplying the information instead of erasing it. Therefore, in order to manage forgetting on the web in a way that aligns with algorithmic memory, one could consider implementing a procedure that goes against the conventional practice of deleting or making content unavailable. This involves employing strategies of obfuscation, which generate misleading, false, or ambiguous data alongside each transaction on the web. In practice, this multiplication of information production aims to impede the meaningful contextualization of data ( Esposito, 2022, 73).

This proliferation of information renders each individual piece of data more marginal, getting lost within the vast volume. Rather than being erased, information becomes invisible due to the obfuscation caused by the excess of data.

As Esposito suggests in her conclusion, the issues arising from the digital ecology need to be addressed from a digital perspective, which entails a shift in the reference frame:

“Algorithms participating in communication can implement, for the first time, the classical insight that it might be possible to reinforce forgetting—not by erasing memories but by multiplying them. This requires a radical change in perspective. It does not solve all the problems of digital memory and of the difficulty in controlling the continuous production of an excess of data, but moves these problems to a different and much more effective level: from the reference frame of individuals to that of communication” (76–77).

## Re-inventing memory

In the article ‘Preservation of Digital Literature: From Stored Memory to Reinvented Memory’ ( 2013) Bouchardon and Bachimont address the issue of preservation in the digital age. This is the opposite problem to the one described above. On the one hand, the persistence and availability of digital data raise the issue of the difficulty of forgetting, exemplified by ‘The Right to be Forgotten’. On the other hand, concerning preservation, the digital era is likely the most fragile and complex context in human history. Compared to a medium like books, which have remained virtually unchanged, allowing us to read works produced hundreds or thousands of years ago, the lifespan of digital works is short. They quickly become obsolete. This is explained by the fact that in the digital medium, content is situated at two different levels.

Bachimont makes a distinction between the "inscription form" and the "restitution form" ( Bachimont, 2007). In the context of printed material, both forms are the same, represented by the printed text. However, in the case of digital media, these two forms are separate due to the intervention of computational processes that mediate between them.

The existence of two forms raises the question of how we define content. Is the content what is stored on the hard drive (the resource), or is it the content that appears on the screen (the rendering)? If we preserve the resource but not the representation, can we still speak of preservation? This problem is evident in the field of digital literature, the topic that Bouchardon and Bachimon address in their article, since a digital literary work is not an object —like a printed book— but fundamentally a process that can only exist through actualization. Bouchardon and Bachimon distinguish four possible strategies for the preservation of digital literature: museology, migration, emulation and description.

The museological approach consists in “preserving contents as they are as well as the tools permitting playability. This way, it is not only the information which is preserved, but the technological environment characteristic of a certain time and content” ( Bouchardon & Bachimont, 2013, 187). Migration involves “updating the technical format of the contents so that they should remain compatible with and adapted to the reading tools available in the current technological environment” (188). In emulation “the contents are not made to evolve. Instead, the reading tools of the old formats are simulated on current environments” (188). Lastly, description, an approach that is “counter-intuitive but the most potent on a theoretical level” consists in “discarding recorded contents in as far as they are incomplete, partial or ill-defined. Therefore, it is better to preserve a description of the content which permits us to reproduce it. The description may concern the reproduction of key elements, of the authors’ intention, and the variable media approach, of the graphic appearance, etc;” ( Bouchardon & Bachimont, 2013, 189–90).

The authors compare the description-based preservation method with the preservation model of classical music. How do we know how to play Baroque music today when we do not have recordings of how it was performed in its time? Thanks to the combination of three elements: score, instrument, and instrumental practice. The score serves as a set of instructions for producing music on an instrument. Organology ensures the preservation of instruments and the techniques involved in their creation. Furthermore, through the practice of music, which involves reading scores and playing instruments, knowledge is continuously taught and passed down. Preservation goes hand in hand with constant usage. In this sense, the concept of music serves as a model for preserving a content that cannot be directly recorded. Instead, it relies on saving resources (the score), a player (the instrument), and a practice (music education). This unique musical solution allows for the preservation of a non-variable description of the performance, even in the absence of the original content.

As Bouchardon and Bachimon point out, this preservation model —which does not preserve the content intact but rather the elements necessary for its reconstruction— is essentially an interpretative act. In this model, preservation is synonymous with (re)invention: “Preserving is keeping intact the interpretability of the work to be able to reinvent it. In other words, preserving is saving the knowledge of its re-invention” ( Bouchardon & Bachimont, 2013, 191).

This model, which the authors argue is "from an anthropological point of view" "more valuable and more authentic than the model of printed media which is a memory of storage" (200) effectively embodies one of the central characteristics of collective memory. Like the ontological dynamism of archives, the dialectic between memory and forgetting in the digital age brings us closer to the functioning of collective memory understood as the continuous evolution, change, and adaptation of representations of the past in the present. What collective memory preserves is not an ‘intact’ past. It does not faithfully preserve the facts, characters, or significant events of that past for a community —which does not imply that the representations of the past it preserves are false. Collective memory re-appropriates, adapts, and re-actualizes the facts of the past, along with their affects and values. Re-invention makes room for the creation of new relationships with the past, that is ultimately, the goal of collective memory.

## Conclusion

The digital revolution has had a significant impact on collective memory, resulting in notable changes. Archives, which were once static repositories, have undergone a transformation and now function as dynamic entities. This shift challenges traditional storage methods and promotes new archiving practices that aim to make information more accessible to everyone.

The digital era has prompted a reversal in the relationship between memory and forgetting. What used to be a demanding endeavor requiring significant resources, as memory was a scarce commodity, now appears as an activity we do not even have to worry about. Our visits, purchases, the number of pages we have read in a book, the photos we take on vacation —everything is automatically recorded and preserved. At the same time, the networked operation of our devices can potentially make this data available beyond our intentions and into an unpredictable future.

Now, forgetting is what appears to be a valuable asset to preserve, something that attempts to be regulated through laws like the ‘Right to be Forgotten’. As Makhortykh (2021) suggests, it is interesting to consider whether the ‘Right to be Forgotten’ could also apply collectively and what the consequences would be. What happens if a perpetrator or a victim requests the deletion of information related to their involvement in atrocities? What would occur if a state implicated in crimes against humanity claimed this right?

The flip side of this process is that, despite the apparent persistence of data on the web, the internet is not an ideal mnemonic medium. On the contrary, the fragility of data, caused by factors such as the rapid obsolescence of storage devices, led early critics of the internet in the mid-90s to warn that we might be living in a ‘dark age’ where records will not be preserved. Various responses have been proposed to address the difficulty of preserving data in the digital age, or more specifically, digitally-born content, where content is not an object but fundamentally a process that can only exists through actualization, with interactivity being an essential part of it.

Among these responses, the most interesting one is the intertwining of preservation with reinvention. The metaphor of the ‘Sappho syndrome’ ^5^ is valid in envisioning a memory appropriate to the digital era. If works disappear to the extent that all that remains are fragments and descriptions made by others, comments on comments, all that is left is to reinvent them. But isn't that what collective memory has always done? Reinventing the past based on its remnants and the narratives others have constructed about it.

Digital media has introduced powerful tools that allow us to observe interactions between actors —both human and non-human— with unprecedented precision and abundance of data. What are the consequences of this for the way we conceive of the social? How can a mode of conceiving the social enable us to capture social memory more faithfully? Tarde's proposal, which considers the articulation between the individual and the collective, the micro and the macro, without favoring one at the expense of the other, can provide us with an appropriate conception of the social for thinking about it in the digital age, as well as refining the shortcomings inherited from Halbwachs.

In sum, the extent of the changes introduced by the digital turn is undeniable. However, does it make sense to categorize digital memory as a completely new and radically different form of memory? Does the digital render the concept of collective memory obsolete? As I hope to have showed, collective memory, conceived as a process that is mediated and remediated by multiple media, with the participation of dynamic communities that perform rather than represent the past, is still valid. Furthermore, not only is it valid, but the digital realm materializes its processual nature —which is inscribed in the very archive— brings to the forefront the role of technological mediation and make visible the associations that produce ‘the collective’ without representing collective memory as a disembodied entity that transcend the individuals who comprise it.

## Data Availability

No data associated with this article
