# Memory in the digital age

**DOI:** 10.12688/openreseurope.16228.2

**Published:** 2024-01-12

**Authors:** Silvana Mandolessi

**Affiliations:** 1 Cultural Studies, KU Leuven Association, Leuven, Flanders, 3000, Belgium

**Keywords:** Digital memory, collective memory, memory studies, forgetting, archive

## Abstract

This article explores the configuration of collective memory under the impact of the digital turn. In recent debates, there has been a marked tendency to interpret ‘digital memory’ as a new type of memory, which is radically different from the traditional conceptualization. Even leading authors in the field claim that the digital revolution implies the end of collective memory. However, I argue that despite the transformations that memory undergoes in the digital age, these changes do not imply a new ontology of memory but rather a materialization of the theoretical claims made by Memory Studies since the field's inception.

To support this hypothesis, I analyze digital memory in relation to three topics: first, I focus on the problematic definition of collective memory to demonstrate how the digital realm allows us to rethink the social nature of memory through a different concept of the social. By contrasting Halbwachs' notion of the social, which forms the basis of memory studies, with the alternative proposal of Gabriel Tarde, I argue that the latter enables us to refine the concept of the ‘collective’ that we have inherited from the founding figure of memory studies. Second, I delve into the new ontology of the digital archive showing how it materializes one of the defining features of collective memory: its mobile, dynamic, and procedural nature. Lastly, I address the inversion of the dialectic between memory and forgetting to highlight the specificity of these practices in the digital environment. I demonstrate how these changes effectively implement, surpassing older technologies, the concept of collective memory as a distributed and dynamic technological process that shapes our shared representations of the past.

## Plain language summary

In everyday language, memory is conceived of as an individual faculty; however, remembering is an eminently social act. Societies elaborate representations of the past that are fundamental to the constitution of their identity. Following this, the field of memory studies has explored the way collective memory, the relationship that a social group establishes with 'its' past, is socially constructed and circulates, as well as its multiple effects on our experience of the present. One of the fundamental insights of memory studies is that memory is always mediated. Different media shape memory in different ways, providing memory with specific affordances and constraints. If the advent of writing in early civilisation radically altered the constitutions of memory cultures, and the invention of print marked a new shift putting memory in circulation in an unprecedented way, the onset and spread of digital media signals the more recent revolution in collective memory and mnemonic communities. This article explores the configuration of collective memory under the impact of the digital turn. Recent debates suggest that ‘digital memory’ is a completely new form of memory that is fundamentally different from traditional memory. I argue that while memory has certainly transformed in the digital age, these changes don’t create a new type of memory but materialize the theoretical claims made by memory studies from its inception. To support this argument, I examine digital memory in three key aspects. First, I discuss the definition of collective memory and how the digital realm allows us to reconsider the social nature of memory. Second, I explore the new nature of digital archives and how they embody the essential characteristics of collective memory: mobility, dynamism, and procedural nature. Lastly, I address the inversion of the dialectic between memory and forgetting to highlight the specificity of these practices in the digital environment.

## Introduction

The impact of the digital turn on our culture is undeniable. It has changed the way we consume and create content, breaking down geographical barriers, and empowering individuals to participate in cultural expression on a global scale. With the rise of social media, digital media has also transformed how we communicate and interact with each other, changing the way we form communities and share ideas. One of the major questions that are systematically asked in the debates on the digital is the scope and stakes of this impact. Is the digital turn a real break with previous cultural frameworks? Or, on the contrary, it is better to think the digital as a remediation of the older media? In this article, I aim to examine the impact of the digital turn on ‘collective memory’ *.* Following Schwartz, collective memory can be defined as “the distribution throughout society of what individuals know, believe, and feel about the past, how they judge the past morally, how closely they identify with it, and how much they are inspired by it as a model for their conduct and identity” ( Schwartz, 2016, 10)

Since what has been called the ‘memory boom’ in the 80s, collective memory has been an object of continuous academic inquiry as well as a constant presence in public debate. Fueled by various factors —including the exceptional scope of violence and wars of the 20th century, changes in media technology, the end of the Cold War, and developments within academia ( Erll, 2011a, 4–5)— the memory boom led to a detailed investigation of the role of memory in the formation of collective identities, its political function, its stability and variability over time and space, its fidelity or distortion of historical events, its ‘insufficiency’ or ‘excess’ ( Connerton, 1989; De Cesari & Rigney, 2014; Levy & Sznaider, 2006; Olick *et al.*, 2011a; Radstone & Schwarz, 2010; Rothberg, 2009; Tota & Hagen, 2016).

In the last 30 years, memory studies not only became institutionalized, as evidenced by the existence of specialized journals and editorial collections, but they also, in some way, became standardized —or at least a part of them— around an approach to the representation of the past that ended up being repetitive and sterile. Assessing the current state of the field and the future of memory studies, Astrid Erll stated,

“With the methodology at hand, memory studies will easily be able to keep generations of scholars busy, charting the mnemonic practices of all ages and places. However, the question arises whether ‘memory’ is thus turning into a mere ‘stencil’, and memory studies into an additive project: we add yet another site of memory, we address yet another historical injustice. While such memory work is for many historical, political and ethical reasons an important activity, memory research finds itself faced with the decisive question of how it envisages its future” ( Erll, 2011b, 4)

Erll's critique of the need to update the conceptual and methodological framework of memory studies is even more evident in the contemporary scenario. Today, it is impossible to understand the shaping and transmission of collective memory without addressing the changes brought about by the digital turn. However, although there is consensus on acknowledging the impact of electronic media —something that would be futile to deny— the extent of these changes is a subject of debate. Is it an evolution or a revolution? Are we witnessing a radical break from pre-digital forms or a cultural continuity where mnemonic practices do not undergo substantial modification?

In the recent debate, the prevailing idea is that digital memory represents a radical change, giving rise to a new form of memory referred to as “algorithmic memory,” “connective memory,” “new memory,” or “the memory of the multitude” ( Blom, 2017; Garde-Hansen *et al.*, 2009; Makhortykh, 2021; Hoskins, 2009; Hoskins, 2011; Hoskins, 2018b). According to these perspectives, digital memory not only implies a different ontology but, in more radical proposals, even entails the “end of collective memory” ( Hoskins, 2018a).

Andrew Hoskins, one of the leading figures in the field of digital memory studies, undoubtedly best represents this position. Hoskins argues that the impact of digitization implies an ontological shift that renders the conceptual vocabulary previously used in memory studies obsolete, especially its central concept, collective memory. In his formulation, digital memory is based on hyperconnectivity, where agency is both technological and human. This marks a key difference from the previous era. Technological agency —the role of algorithms, the dynamism of archives, the quantity and scope of available information— leads to fragmentation, dispersion, unpredictability, and a loss of control over memory:

“temporary technological arrangements […] open up a new kind of social articulation of digital memory: immediate, hyperconnective, remote, but whose parameters (size and duration) are unpredictable as they have a feature that the traditional idea of the social does necessarily possess, that they are archival. And it is through shadow archives that the individual and the social are inescapably entwined and also suspended, in a kind of extratemporal and extraspatial existence, and made contingent on the potential of their reinvigoration or rediscovery (emergence)” ( Hoskins, 2018a, 93).

In Hoskins’ pessimistic view, the digital realm radically destabilizes the temporal and spatial boundaries of collective memory. A central factor of destabilization is the inherently archival nature of digital media:

“The notion of the archive as static is replaced by the much more fluid temporalities and dynamics of ‘permanent data transfer’ ( Ernst, 2018) defining the new memory ecology. The archives […] are rendered fluid through their hyperconnectivity” ( Hoskins, 2018a, 87).

While archives in the previous era ensured the stability of collective memory, anchored in both time and space, the archive in the digital era possesses its own temporality that we do not control. As Hoskins claims,

“Today, the connective turn has rendered most communications, everyday and exceptional, as potentially part of shadow archives, in which sharing without sharing has no limits. Once media became potentially infinite (in time and space), then duration of our multiple presences-in-the-world became uncertain and uncontrollable. Collective memory has its limits. Memory of the multitude does not” ( Hoskins, 2018a, 106).

And the same happens with temporal anchoring, as Hoskins asserts, “the archive today is the inverted archive, which challenges the significance of place and location. Clearly, place and location are still important, but in many ways they are transcended, if not displaced, by connectivity, time and algorithmic search” ( Hoskins & Halstead, 2021, 678).

The second factor that destabilizes collective memory lies in the digital practices of individuals —what we *do* in the digital environment. Hoskins describes these practices as “compulsive”, once again highlighting the lack of control, in which individuals seem to be engaged in frenetic activity, “posting, linking, liking, recording, swiping, scrolling, forwarding, etc., digital media content” […] constituting a new coercive multitude that does not debate but rather digitally emotes (as in via emoticons) ( Hoskins, 2018b, 2).

While the participatory nature of digital media has often been interpreted as a democratization of collective memory, in this case, digital practices lack democratizing potential and instead result in a radical dispersion and fragmentation of collective memory. Hoskins then proposes the term “the memory of the multitude” to replace the concept of collective memory. Unlike the stable or organic communities of the pre-digital era, the multitude is characterized by its diffusion, dispersion, provisional nature, and perpetual becoming. Inspired by the work of Hard and Negri, and Virno, “the multitude is an internally different, multiple social subject whose constitution and action are based not on identity or unity (or, much less, indifference) but on what it has in common”. The individual, placed at the center of the media ecology, connects with other individuals and forms “aggregates” or “masses”. In Merrin’s words, “these masses are fluid, flexible, variable, ephemeral, constructed by our voluntary participation and continually evolving, and we are involved in many of these at once. What networked computing realizes, therefore, is ‘the multitude’” (93).

While Hoskins asserts that the multitude as a collective subject is an organic community of a different kind, “not characterized by stability and homogeneity, but rather by diffusion, dispersal, and emergence”, his final assessment accentuates the vulnerability of this social formation. Hoskins even questions whether the multitude might give rise to a society without memory.

“What prospects are there, then, for the translation of the digital present into some kind of usable past? Is it that the multitude is the basis of a society without memory? As the social and cultural ‘frameworks’ of remembering have moved inside the machine and inside us, the multitude is increasingly vulnerable to the vagaries of the network […] In sum, the multitude is the impossible crowd: attracted by the immediacy, continuity and sociability of its hyperconnective relations, and yet, with diminishing means to ever escape its digital shadow” ( Hoskins, 2018a, 105–6).

These words ultimately condemn the multitude as a collective subject of meaningful memory. Digital memory does not represent a new phase in the development of our relationship with the past but the “end of collective memory”. Are we indeed facing the end of collective memory? Should we abandon the conceptual framework and insights gained along the way, closing the debates that have occupied us by deeming them irrelevant in the contemporary setting? I would claim the opposite. Digital memory is not a new form of memory, and far from representing the end of collective memory, it materializes and puts into practice the characteristics with which we have defined collective memory since the inception of the field. In fact, we could argue that what we have always advocated about collective memory finds its true realization in the digital era.

My argument is that Hoskins’ ‘pessimistic’ view is derived from a conception of collective memory that emphasizes its permanence and stability. In the traditional conception, the past provides coherence and certainty. The title of the introduction to the influential volume *Digital Memory Studies* is precisely “The restless past”, a past that has been (fatally) deprived of its stability, and, therefore, its ability to provide coherence to the present —and the future. In Hoskins’s words:

“Memory has been lost to the hyperconnective illusion of an open access world of the availability, accessibility, and reproduceability of the past. I say ‘illusion’ as our submergence in post-scarcity culture has also elided what is really at stake here: the loss of the security of vision that the past once afforded (a clear sense of the why of the difference) and a slippage of the grasp of what effect all our current entanglements with media will have on remembering and forgetting. The past has been stripped of its once retrospective coherence and stability, entangled in today’s melee of uncertainty” ( Hoskins, 2018b, 5–6).

The emphasis on coherence and stability as central features of collective memory is evident in the factors that Hoskins analyzes as indicators of the change between the previous era and the present: the ontology of the archive —which is no longer static but dynamic; technological agency —now dominant; the inversion of the dialectic between memory and forgetting —previously forgetting was the norm, while now it is the exception; the transformation from an organic community into ‘the multitude’. From these changes arises the obsolescence of the concept of collective memory, which essentially designates a coherent, stable, and bounded memory and, therefore, would no longer be useful for describing digital memory.

Overall, I agree with the changes that Hoskins highlights as central to understanding the current state of collective memory under the impact of the digital. Factors like the new ontology of the archive are indeed central to the debate on digital memory and have been extensively theorized in fundamental works by scholars like Wolfgang Ernst ( Ernst, 2013), Røssaak ( Røssaak, 2010), Blom ( Blom *et al.*, 2017) or Brügger ( Brügger, 2018). However, I argue that these changes do not represent a new ontology or the end of collective memory but rather that this new phase constitutes an update and materialization of the characteristics by which we have always defined it.

My position is that the primary feature of collective memory does not lie in its stability but in its dynamism. This does not imply denying continuity or coherence —always provisional but effective— of collective representations of the past but rather highlighting that dynamism or variability is what underlies reflections on collective memory. Maurice Halbwachs’ theory of the “social frameworks of memory” is, in fact, a response to the variability that Henri Bergson observed regarding individual memory. Halbwachs discusses Bergson —as I will develop in the following section— with a theory that explains this dynamism based on changing social frameworks. At its core, change and not permanence is the defining feature of memory. Hence my argument that collective memory —understood as essentially dynamic— does not come to an end with the ‘digital turn’ but, on the contrary, is enhanced.

Starting from the essential dynamism of collective memory, in what follows I will examine digital memory in relation to three topics.

First, I will focus on the definition of collective memory to demonstrate how the digital realm allows us to rethink the social nature of memory through a different concept of the social.

Maurice Halbwachs laid the foundation in the field of memory studies by theorizing the existence of a collective memory —shared representations of a common past— that guides our understanding of the present. However, despite the consensus that Halbwachs occupies a foundational place, the concept of collective memory inherited from Halbwachs has been criticized for its imprecision, stemming from its disembodied, homogeneous, and abstract conceptualization of the social. The digital era represents an exceptional opportunity to rethink the conception of the social inherited from Halbwachs because it reveals that an organic, homogeneous, and stable community associated with a specific territory and defined in generational terms does not adequately describe ‘the social’ as it manifests today. Does this mean that we need to abandon the term collective memory? No. The term collective memory defines the social, cultural and political dimension of shared memory that goes beyond the individual. I do not see any reason why collective memory should be a concept limited to the era of mass media, rather than referring broadly to this shared dimension. What is necessary is to review whether the way Halbwachs defined the social is still valid. It is not just a matter of stating that this concept of the social was valid when Halbwachs theorized and is no longer valid today, but of exploring what alternative conceptualizations of the social existed at that time and which Halbwachs did not choose. We know that Halbwachs’ theory is indebted to Emile Durkheim in his view of the social. However, alongside Durkheim, other sociologists proposed a different way of conceiving the social. Among them, Gabriel Tarde stands out, who has been rediscovered in recent years, and who Bruno Latour has called a precursor of digital theory. By contrasting Halbwachs’ notion of the social, which forms the basis of memory studies, with the alternative proposal of Gabriel Tarde, I argue that the latter enables us to refine the concept of the ‘collective’ that we have inherited from the founding figure of memory studies.

The second topic that I address is the new ontology of the digital archive. Following the proposed hypothesis, my argument is that the new dynamism of the archive does not threaten the stability or coherence of collective memory. On the contrary, the new ontology of ‘the archive in motion’ (Røssaak) represents the utopia of an archive that remains and changes simultaneously. An archive that, instead of remaining static —outside of time— inscribes the drift of time on its very surface. In this sense, rather than representing a threat, the digital archive is the realization of the desire to possess a device capable of recording the variability of memory and evolving alongside it.

Finally, I address the dialectic between memory and forgetting. One of the key points of debate in the field of digital memory is the new balance —or lack thereof— in the relationship between memory and forgetting. Theses regarding the preeminence of one over the other are contradictory, and in many cases, apocalyptic. On one hand, it is argued that in the digital age, forgetting does not exist, and we are constantly threatened by the possibility of the involuntary reemergence of that which we would prefer to remain in the past. Regulations such as the ‘Right to be forgotten’ (referred to in the third section) strive to guarantee that right, attempting to correct the ‘excess’ of memory fostered by digital media. On the other hand, it is pointed out that digital memory, far from being the utopia of perfect memory, is fragile, vulnerable, inconsistent, ephemeral. In this sense, the digital represents a radical threat to the very possibility of remembering. If the supports that house traces of the past disappear, if all that is solid melts into air—or rather dissolves in the broken links of web pages and obsolete devices—how can memory be ensured? I argue that, far from apocalyptic visions, beyond the truth value they hold, digital memory invites us to rethink new, dynamic forms of memory and forgetting.

Just as memory has traversed different phases following technological changes, ranging from orality, the emergence of writing, the era of mass media, to the digital age, accompanied by the various metaphors that define it, forgetting can also be thought of as variable and dependent on the evolution of media. In this sense, the suggestion by Hoskins to reconsider forgetting is undoubtedly indispensable. How can we conceive a notion of memory and a notion of forgetting suitable for the digital era? In this section, I draw on the reflections of Esposito and Bouchardon, who suggest particular forms of memory and forgetting in the digital age.

When I state that digital memory is not new, I do not mean to say that memory has not undergone changes under the impact of the digital realm—which would be absurd to claim. On the contrary, the changes are profound and they affect both the actors involved, as well as the objects and practices. What I propose is that these changes, instead of constituting a new memory, actually materialize or implement the essential characteristics that define collective memory. What digital memory offers us is a memory that can now align more perfectly with its own definition, bridging the gaps between theory and practice. It also allows us to reconsider problematic aspects in theory under a new light.

## Rethinking the social

In 1925, Maurice Halbwachs published *Social Frameworks of Memory* ( *Les cadres sociaux de la mémoire*), a book in which he advanced the concept of ‘collective memory’. While Halbwachs was not the only one to address the social nature of memory at that time —Aby Warburg is often cited as representing an alternative genealogy in the field— the current concept of collective memory derives from his reflections ^1^.

Halbwachs's exploration of memory integrated perspectives from two influential figures in late nineteenth-century France, philosopher Henri Bergson and sociologist Emile Durkheim ( Olick *et al.*, 2011a, 30–36). Bergson conducted a radical philosophical analysis of the experience of time, emphasizing the central role of memory within it. He challenged the notion of memory as a passive storage mechanism and instead characterized remembering as an active process. Bergson's exploration of memory brought to Halbwachs's attention the distinction between objective (often transcendental) and subjective perceptions of the past. Against the uniform time of the clock, individual memory was essentially variable and dynamic, vividly capturing short periods while leaving longer periods only vaguely outlined.

Durkheim agreed with Bergson in rejecting transcendentalist explanations of time but, unlike Bergson, Durkheim attributed the variability of perceptual categories not to subjective experiences but to differences among various forms of social organization. According to Durkheim, groups share ‘collective representations’ —narratives, symbols, meanings— that constitute the group but not need to be shared by all members of the group. This is the reason Durkheimian approaches has been often criticized “for being radically anti-individualist, conceptualizing society in disembodied terms, as an entity existing in and of itself, over and above the individuals who comprise it” ( Olick *et al.*, 2011a, 30). Another important characteristic, as Olick, Vinitzky-Seroussi, and Levy highlight, is “the tendency to assume, without justification, that these societies constituted by ‘collective representations’ are homogenous entities. Consequently, a Durkheimian approach to collective memory can lead us to attribute a single collective memory or a set of memories to entire, well-defined societies” (33). As J. Olick, Vinitzky-Seroussi, and Levy summarize, “by connecting cognitive order (time perception) with social order (division of labor), Durkheim thus provided for Halbwachs a sociological framework for studying the variability of memory raised by Bergson” ( Olick *et al.*, 2011a, 31).

For Halbwachs, society is composed of different groups —the family, social classes, religious groups. Each of these groups has its own distinctive memories that give the group continuity, coherence, and stability over time. The group’s own memory comprises a shared body of concerns and ideas and is sufficiently general and even impersonal to keep its meaning beyond the variations that may arise from the perspectives of each member.

Halbwachs says:

“Each man is immersed successively or simultaneously in several groups. Moreover, each group is confined in space and time. Each has its own original collective memory, keeping alive for a time important remembrances; the smaller the group, the greater the interest members have in these events […] In such milieus all persons think and remember in common. Each has his own perspective, but each is connected so closely to everyone else that, if his remembrances become distorted, he need only place himself in the viewpoint of others to rectify them” ( Halbwachs, 1980, 78)

Collective memory then appears as an exclusive property of the group —exclusive in the sense that individual differences never seem to threaten the homogeneity of the ‘common to all’.

It is this conception of collective memory as an abstract, disembodied, and homogeneous entity that has been criticized for not providing an adequate framework to capture the social nature of remembrance. In the current digital environment, this inadequacy becomes even more apparent.

 Following a Durkheimian approach, the term ‘collective’, in the classical theory of Halbwachs, refers to social groups with a dynamic but stable identity. Even if an individual can be part of several groups, they appear as clear-cut formations, unified by a shared identity, a set of shared values, and attached to a territory. Today, on the contrary, the social —as in social media— is the product of a social engineering driven by the entanglement of algorithmic forces, shared digital values, and participatory practices. The meaning of ‘social’ hence encompasses both (human) connectedness and (automated) connectivity, since social media are inevitably automated systems that engineer and manipulate connections, by coding relationships between people, things, and ideas into algorithms ( Van Dijck, 2013, 12). As Latour (2005) puts it, the social is not a thing or domain, not an explicative category but precisely what needs explaining. This becomes even more acute in the digital era, in which the social is not a fact but a doing, performed through digital connections ( Bucher, 2015). As Blom wonders about the suitability of the concept of collective memory in the digital age,

“But what if the material frameworks of memory seem to lack the type of stability and durability that confer identity on things? What is a society’s self-image if this image may be the object of instantaneous erasure, dispersal through multiple relays or information overflow, or transmutation through dynamic feedback circuits? What is society if its memory images are perhaps not even representations?” ( Blom, 2017, 14).

The question is: what conception of the social would allow us to understand the collective nature of memory in the digital era? Gabriel Tarde's theory may provide us with an answer. ^2^ It can constitute an alternative for rethinking the social ontology inherited from Durkheim, which forms the basis of Halbwachs’ conception.

## Gabriel Tarde versus Emile Durkheim

The nineteenth-century sociologist Gabriel Tarde (1843–1904) has experienced a sort of revival in recent years. His theories have been revealed as inspiring or precursors to the theories of Foucault, Deleuze, or Latour, who has described Tarde as “the forgotten grandfather of Actor-Network Theory” ( Candea, 2010, 1).

During the 1890s, debates surrounding the object, methods, and the very definition of sociology multiplied, and increasingly, these sociological debates came to gravitate around the crucial contrast between Tarde’s and Durkheim’s answers to these questions. The disputes between Tarde and Durkheim —which can be traced in their respective publications, where one comments on and refutes the other – culminate in 1903 in an epic debate between the two thinkers at the École des hautes études sociales ( Candea, 2010, 5). As we know, despite the recognition Tarde enjoyed in his time, it was Durkheim who ultimately took center stage. But, Candea wonders

“What if Durkheimian sociology had had, from the very beginning, a thoughtful and vocal opponent; one who queried the ‘thingness’ of the social and the holistic, bounded nature of societies and human groups […] one who foregrounded imitations, oppositions and inventions where Durkheim saw conformism to a rule as the key component of the social; one who had already found a way to dissolve the linked contrasts between individual and society, micro and macro, agency and structure, freedom and constraint —Durkheim’s main (and for many, troublesome) legacy to twentieth-century social science?” ( Candea, 2010, 1)

Durkheim and Tarde differ in the role they assign to individuals in relation to society. While for Durkheim, social facts or collective tendencies impose themselves on individuals, Tarde places the individual at the center, and it is through events of association —and the laws of imitation —that the social is constituted.

A central point of disagreement between Durkheim and Tarde lies in the definition of the social fact and in the force with which they impose themselves on individuals. According to Durkheim, ‘collective tendencies’ have their own existence and depend on forces external to individuals. Durkheim compares them to cosmic forces, and like them, their existence is demonstrated by the uniformity of their effects.

For Durkheim, a social fact is identifiable by the power of external coercion it exerts or is capable of exerting on individuals. In fact, a mode of behavior that exists outside the consciousness of individuals only becomes generalized by exerting pressure on them. Therefore, two central features characterize a social fact for Durkheim: the first is that it exists independently of its individual expressions; the second, no less important, is its coercive nature.

If, for Durkheim, social phenomena are explained through coercion, for Tarde, on the other hand, they find their foundation in imitation. For Tarde, all social facts are the product of individual inventions propagated by imitation. Any belief and any practice would have at its origin an original idea born from an individual brain. Whether they are considered useful or because their author is invested with authority, other members of society adopt them, and once generalized, the invention ceases to be an individual phenomenon and becomes a collective phenomenon. Imitation is, therefore, the key. Emerging from the individual and through the laws of propagation, the fact becomes social.

Durkheim criticizes the idea of imitation, arguing that a movement repeated by all individuals does not constitute a social fact for this reason. “What constitutes a social fact”, —he answers— “is a belief, tendency, or practice of the group taken collectively, which is something else entirely than the form it may assume when it is refracted through individuals” ( Durkheim, 1894, 54).

Then, there is a fundamental disagreement in the relation between the whole and the parts. For Durkheim, the whole is always more and essentially different from the form it acquires in the refractions of individuals. On the contrary, for Tarde, “how could it be refracted before existing, and how could it exist, let us speak intelligibly, outside of all individuals?” It is Tarde himself who presents his theory in the following passage, and it is why it is convenient to quote him extensively:

“The truth is that a social thing, whatever it might be [...] devolves and passes on, not from the social group collectively to the individual, but rather from one individual [...] to another individual, and that, in the passage of one mind into another mind, it is refracted. The sum of these refractions, from the initial impulse of an inventor, a discoverer, an innovator or modifier, whoever it might be, unknown or illustrious, is the entire reality of a social thing at a given moment; a reality which is constantly changing, just like any other reality, through imperceptible nuances; this does not prevent a collectivity from emerging out of these individual varieties, an almost unchanging [ *constante*] collectivity, which immediately strikes the eye and gives rise to Mr Durkheim’s ontological illusion” ( Tarde, 1895, 66–67).

In summary, Tarde does not deny the existence of a collectivity, but he does not place that collectivity above and beyond individual expressions. The collective emerges from a generalization of individual varieties, and while it may acquire consistency and stability, this stability is provisional —even if it can produce the ontological illusion that Tarde accuses Durkheim of. Tarde’s view is, unlike Durkheim’s, essentially dynamic. He describes the social world as the result of events of association in which contingency plays a significant role. Thus, while Durkheim is interested in studying ‘social facts as things’, as entities that exist beyond individuals, Tarde chooses to focus on ‘things as social facts’, which implies assigning a social character to all entities. “This relocation”, assert Harvey and Venkatesan “has radical potential in relation to established, positivist social science, for it foregrounds the processual and captures the importance of relational dynamics of becoming, the open-endedness of all things, the potential of all things to transform through their inherent situated relationality” ( Harvey & Venkatesan, 2010, 129–30).

Tarde’s view is a conception of the social that is not limited to human relationships. Not only are human beings social, but social behavior is inherent in all phenomena in the universe, from atoms, nations, cells, and bacteria to plants and animals. As Tarde remarks, “every thing is a society and every phenomenon a social fact” ( Tarde, 1999, 58). Society is thus not an exclusive trait of humans but extends far beyond to encompass all kind of phenomena.

Why does Tarde’s conception of the social become relevant in the digital age? And what are the implications of this conception for collective memory?

Tarde’s conception of the social becomes relevant in the digital age at least for three reasons:

First, Tarde’s idea that the social is constituted through relationality resonates in the digital culture, where connectivity is a defining feature. In the digital era, online interactions, social networks, and constant connectivity among individuals reflect the notion of social entities that Tarde proposed.

Second, Tarde’s vision, which does not limit the social to human relationships, is particularly relevant in the digital era, where technology plays a central role. Relationality extends to algorithmic connections and the interaction between humans and technology. This provides a broader and more comprehensive perspective on sociability in the digital context.

Third, Tarde’s thesis of imitation serves as a model for understanding the spread of ideas, behaviors, and emotions in digital networks. In an environment where information spreads rapidly through online platforms, Tarde’s notion of imitation provides a lens for analyzing how trends disseminate in the digital space. A book like *Virality: Contagion Theory in the Age of Networks (2012)* by Tony Sampson, which draws on the social epidemiology developed by Tarde, serves as an example.

The rediscovery of Gabriel Tarde in recent decades is framed within the preeminence that several concepts have gained in the social sciences and humanities. These concepts emphasize that social phenomena are complex, procedural, indeterminate, relational, and constantly open to the effects of contiguous processes. This epistemological shift shares a common ontology that erases the differences between the social and the natural, the mind and the body, the cognitive and the affective. It is grounded in concepts such as assemblage, flow, emergence, becoming, relationality, the machinic, the inventive, the event, the virtual, and the informational ( Blackman & Venn, 2010).

Precisely in the digital environment, the distinction between the human component and the technological component dissolves, and what emerges, instead, is a “human-data assemblage” ( Lupton, 2016). By not restricting the social to human actors, Tarde’s theory allows us to explore collective memory as a process encompassing both (human) connectedness and (automated) connectivity. The role of algorithms in encoding the relationships between people, things, and ideas is essential for understanding how shared representations of the past are formed and transmitted in the contemporary landscape. Algorithms play a crucial role in shaping the way information is curated, filtered, and presented to individuals within digital platforms and networks. They influence what content is prioritized, recommended, and made visible to users, thereby shaping their understanding and perception of historical events, cultural narratives, and collective memory. Understanding the role of algorithms in the encoding of relationships and the formation of shared representations of the past is crucial for critically analyzing and navigating the contemporary digital landscape. It prompts us to consider the potential biases, limitations, and power dynamics inherent in algorithmic systems and their impact on collective memory. Based on a conception of collective memory indebted to Durkheim’s paradigm, this exploration is simply impossible.

There is a fundamental difference between Durkheim and Tarde that revolves around attention to detail, as well as the significance given to contingency. As Harvey and Venkatesan argue, for Durkheim, “the details of specific interactions were necessarily subordinated to the ‘bigger’ picture. Contingency, error, uncertainty were simply not relevant in his view of the social, where the whole was necessarily more than the sum of its parts and thus beyond the detail. But for Tarde, as for many contemporary ethnographers, the detail is not approached as less than the whole, for it is through attention to the detail that we can find different kinds of collectivity in formation” ( Harvey & Venkatesan, 2010, 130). In the writings of Halbwachs, groups appeared as organic entities in which the individual was subsumed. Even though an individual could be part of various groups and move between them, this mobility did not imply any contradiction or friction. Upon ‘entering’ a group, the individual adopted the collective point of view, and any individual variation became subsumed in the whole. What we call detail, therefore, is nothing less than the specificity of individual variations in the interactions that shape a community.

This is precisely what digital technologies design and make visible: the associations that produce “the collective” without subsuming them under a ‘structure’ a ‘social fact’, or a ‘collective representation’. Following Tarde’s proposal makes it possible to overcome the abstract and disembodied nature that the notion of collective memory had in Halbwachs. Viewed from a Tardean framework, the collective nature of memory is not a representation of the group that imposes itself from the outside on individuals. Instead, it is the result —always provisional and contingent, in other words, dynamic—of individual narratives that connect and integrate within a broader context that gives them meaning.

If, as Tarde proposes, the central mechanism that underlies sociality is imitation, it is through the propagation of ideas, behaviors, and images that the social is constituted. It is then —in this generalization— that we can speak of collectivity. The social, or collective, character of memory does not necessitate the consistency, stability, and homogeneity proclaimed by Halbwachs. On the contrary, contingency and variability are essential traits. Contingency and variability are essential to explain the dynamic nature of collective memory. The dynamic character is not supported, as Halbwachs argued, in the different social frameworks, in the variety of the groups we are part of, or in the evolution of the groups we are part of. The dynamic character is based on continuous processes of imitation, on the propagation of representations of the past that give rise to a provisional aggregate —a collective— that then dissolves again, fades, decreases in intensity, or stops. It is the conception of the social that harbors in Tarde a provisional, contingent, malleable character —not a fact, a structure, a law, but a becoming. A conception of the social that, shaped by human and non-human actors, a ‘human-data assemblage’, allows us to rethink the collective nature of memory in the digital age.

## The archive in motion

In this section I explore the emerging ontology of the digital archive. In line with the proposed hypothesis, I contend that the newfound dynamism within the archive does not pose a threat to the stability or coherence of collective memory. On the contrary, the novel ontology of the archive embodies the ideal of an archive that both endures and transforms concurrently.

In theorizations of memory, the archive has arguably been the most powerful and enduring metaphor. Well-known analogies of memory, such as Plato’s wax seal or table, the loci of classical art or Freud’s mystic writing-pad, rely on the “imprints-on-a-substrate paradigm”, an imagery that conceives memory as a content preserved in a specific place —whether it's the brain, the library, or the screen ( Burton, 2008, 322). The trait that defines the archive in these metaphors is stability. The archive extracts something from the flow of time and safeguard it from decay, preserving it 'intact' for the future.

Clear examples of this can be found in well-known theories on collective memory, such as the opposition between ‘canon’ and ‘archive’ proposed by Aleida Assmann. For Assmann, “the institution of the archive is part of cultural memory in the passive dimension of preservation” ( Assmann, 2008, 103). The knowledge saved in the archive is inert, “it is stored and potentially available, but it is not interpreted” (103). While the archive refers to the passive dimension of cultural memory, the canon “stand for the active working of memory of a society that defines and supports the cultural identity of a group” ( Assmann, 2008, 106). In a similar vein, Diana Taylor opposes two epistemological systems, coded in the dichotomy ‘archive/repertoire’. ‘Archival’ memory exists as documents, maps, literary texts, letters, archaeological remains, bones, videos, films, and all those items supposedly resistant to change. The repertoire, on the other hand, enacts embodied memory and encompasses mnemonic practices such as performances, gestures, orality, movement, dance, or singing ( Taylor, 2003).

In the digital age the static archive is replaced by an “archive in motion” ( Røssaak, 2010). The archive, conceived as the guarantor of the stability of collective memory, undergoes an ontological change characterized by dynamism and mobility. This dynamism is evident in different domains and levels, involving diverse topics such as techno-mathematical operations governing data transfer, access to previously unavailable materials, and the emergence of new archival practices carried out by amateur communities.

The complexity of the debate surrounding the relationship between the archive and digital technologies lies in the fact that, as Taylor argues, archive simultaneously refers to a place, an object, and a practice.

“An archive is simultaneously an authorized place (the physical or digital site housing collections), a thing/object (or collection of things —the historical records and unique or representative objects marked for inclusion), and a practice (the logic of selection, organization, access, and preservation over time that deems certain objects “archivable”). Place, thing, and practice function in a mutually sustaining way” ( Taylor, 2012).

The changes experienced by the archive in the digital era are observed in the three domains.

Firstly, the archive as thing/object. What is stored in an archive can be any object, ranging from the text of a law, a literary work, a painting, the recording of a musical score, to an Aztec’s mask. All these tangible and material things are transformed into the logic of zeroes and ones. Although digital technologies may speak the language of storage and containment, as Blom argues, in digital media “nothing is stored but code: the mere potential for generating an image of a certain material composite again and again by means of numerical constellations ( Blom, 2017, 12). Blom emphasizes mobility, asserting that “once the archive is based on networked data circulation, its emphatic form dissolves into the coding and protocol layer, into electronic circuits or data flow” (13).

Objects in the archive have, of course, always circulated. In fact, the purpose of an archive is not only to preserve documents but also to make them available in a potential future. The difference is that preservation now relies on inherent dynamism, as the conservation of the object in the logic of zeros and bytes involves its translation into protocols and codes that are then updated again when we request the image or text to read it through the interface. The dynamism of digital objects lies in these algorithmic operations formed by constant regenerations, updates and affordances. Archives are “dynamic living systems, constantly transformed and updated” ( Røssaak, 2017, 202–4). Or as Blom puts it: “The conflation of memory with storage is, in other words, undermined by a technical emphasis on dynamic processes of memorizing. To the extent that computer memory exists, it is essentially activity; virtual as well as actual, and its images are electronic events ( Blom, 2017, 12).

Secondly, the archive as place. The spatial arrangement of an archive within a public building has been replaced by the ‘placelessness’ of documents distributed along multiple sites. Digital memories are often perceived as 'placeless', meaning that one notable change brought about by digital media is the diminished connection to a specific location. Under this perspective, memories in the pre-digital era were strongly tied to physical spaces, which played a crucial role in their interpretation. However, in the present era, this connection fades away, leading to memories that are fluid, detached, fragmented, and omnipresent, constantly shifting without precise spatial references. The loss of a meaningful bond with place connotes, in this imaginary, a loss of meaning, given the centrality that the category of place plays in the shaping, preservation, and transmission of collective memory. The idea of placelessness is linked to the changing traits of the archive and the database as the privileged cultural forms of the digital era. The notion of the traditional archive as static, selective, organised, and restricted to a particular place, has been replaced by a dynamic, non-selective, and multimedia online archive, whose logic is fluidity and ubiquity, and which is always ‘in becoming’ ( Mandolessi, 2021)

However, this imaginary has been questioned by highlighting how digital memory practices actively engage in place-making, rather than succumbing to the logic of hyper-connectivity and all the attributes upon which it is predicated ( Mandolessi, 2021). Rather than deterritorizaling memories, digital archives become key tools for new engagements with place (for different approaches on the issue of digital memory and place see the Special Issue “Locating “Placeless” Memories: The Role of Place in Digital Constructions of Memory and Identity”, edited by Huw Halstead, Memory Studies 14.3 (2021).

Thirdly, the archive as a practice. The practice of archiving involves the selection and organization of objects that will be stored in the archive. The authority of experts is crucial, as the decision to archive certain documents and discard others grants them a legitimization that they do not possess prior to their entry into the archive. In this sense, archivization is performative, its operations of selection and organization, as Derrida claims “produces as much as it records the event” ( Derrida, 1996, 17). The importance of selection is emphasized by Ketelaar, who coined the term ‘archivalization’ to denote a phase prior to the archiving process, meaning “the conscious or unconscious choice (determined by social and cultural factors) to consider something worth archiving. Archivalization precedes archiving” ( Ketelaar, 2001, 133)

The digital has radically altered the phase of ‘archivalization’ in at least two senses. Digital media are inherently archival, meaning that even if we do not consciously decide to archive specific content, it will be automatically stored. For example, our tweets or posts on social media platforms like Twitter or Instagram, the locations we visit through geo-tagging, or our selections on online shopping platforms like Amazon. There are no experts behind the scenes determining which content is worthy of archiving; instead, algorithms are designed to collect and process data. This automation is closely tied to the idea of the Internet as a ‘perfect memory machine’ capable of recording and preserving everything. The persistence of data challenges our right or desire to forget, to leave certain things in the past (which I will address in the next section). The lack of selection in automated storage seems to complicate matters rather than offering a solution to the scarcity of memory.

However, changes in archival practices not only involve algorithms recording information with or without our consent but also encompass alternative selection criteria that go beyond traditional notions of expertise. In the digital era, archives can be constructed by anyone who believes that certain materials are worth archiving.

In her book *Rogue Archives: Digital Cultural Memory and Media Fandom* ( 2016), De Kosnik extensively explores the work of technovolunteer archivists. These ‘rogue archives’ are established and maintained by fans with the aim of preserving and sharing cultural artifacts that are often neglected, banned, or considered outside the mainstream cultural canon. The efforts of these technovolunteers play a crucial role in curating and safeguarding the cultural memory of marginalized groups, rather than relying solely on the assumed archival nature of the internet. These archives possess a democratizing potential that challenges the criteria upheld by official institutions regarding what should be preserved or not. This expansion of value in determining preservation challenges the established norms and opens up possibilities for a more inclusive approach to archiving. Moreover, rogue archives not only enable the preservation of materials that would otherwise be lost but also fundamentally transform the mnemonic function of the archive. According to De Kosnik, the archive, which was traditionally seen as a record of cultural production, now becomes a source of cultural production itself. She introduces the term “rogue memory” to signify this shift from preservation to creativity.

Returning to the question posed in the introduction: In what way does the new ontology of the archive materialize the concept of collective memory?

Collective memory is defined as a practice, an active process that interprets the past in light of the present. However, the metaphor of the archive pointed to a static content, those objects, traces, and remnants of the past that could potentially be activated but also remain inert. This potentiality gave the archive a liminal or ambiguous status. If we define memory as an active process, in what sense can we speak of the archive as a ‘passive dimension of memory’? The new ontology of the digital archive, an archive in motion, brings together potentiality and actualization. In the digital era, practices are inscribed in the archive, which no longer functions as a mere passive container or collection of traces of the past but as a dynamic entity. The archive becomes the site in which the traces of the past are continuously interpreted, updated, and collectively reappropriated in an ongoing process.

## The inversion of the dialectics between memory and forgetting

In principle, collective memory refers to any shared representation of the past by a social group. However, the meaning of memory that dominates the field of memory studies has a more restricted character. It is a memory of violence, atrocity, and human right abuses ( Huyssen, 2011; Levy & Sznaider, 2006). This focus on memory centered on violence is deeply linked to the emergence and globalization of a powerful discourse on human rights, which is materialized in the adoption of the Universal Declaration of Human Rights in 1948. In this foundational text, memory holds a central place as an instrument to promote the defense of human rights, a role summarized in the ‘duty to remember.’ There are two underlying assumptions regarding the ethical and moral responsibility to remember that form the core of the relationship between memory and human rights: The first assumption is that acknowledging human rights abuses and honoring victims through memory represents the ethically correct and necessary response to violence. The second assumption further intertwines memory with human rights: the remembrance of past violence is considered one of the most effective measures to prevent future violence ( Sodaro, 2018). The link between memory and human rights explains the positive role assigned to the act of remembering and the corresponding stigmatization of forgetting. This also helps to explain why, in comparison to the extensive research on the various ways in which societies remember, there is limited focus on how societies forget. In general terms, forgetting has been viewed as something to be combated rather than as an object that requires scrutiny. One notable exception is Paul Connerton's *Seven Types of Forgetting* ( 2008) and *How Societies Forget* ( 2009). Connerton does not stigmatize forgetting. On the contrary, he demonstrates the variety and complexity of the mechanisms involved in the act of forgetting and how forgetting can be necessary, for various reasons and in different contexts, for political and social life.

This lack of interest in forgetting as an object of study —its forms, its actors, its means, and its value— is currently being reversed, becoming central in the debate on digital memory. The value attributed to forgetting is also being inverted. Beyond memory associated with human rights, where the ‘duty to remember’ prevails, the question about the function and benefits of forgetting is being brought back into the spotlight. In the digital age, the dialectic between memory and forgetting has been reversed As Mayer-Schönberger claims “Quite obviously, remembering has become the norm, and forgetting the exception” ^3^ ( Mayer-Schönberger, 2009, 52). We need to forget. To forget what? To forget how? And why? The debate can be structured around these questions.

In the debate about memory in the digital era, the essential function of forgetting as an individual and social mechanism is affirmed. The importance of forgetting has been even ratified by a legal framework known as ‘The Right to be Forgotten’ ^4^. ‘The Right to be Forgotten’ is a legal concept that refers to an individual's right to request the removal or deletion of personal information from online platforms and search engine results. It recognizes the importance of privacy and allows individuals to have control over their personal data and its availability on the internet.

Despite the existence of a legal framework that guarantees the right to be forgotten, the question of whether it is possible to forget —or be forgotten— in a digital environment remains. Is it true that forgetting becomes impossible in the digital era? Or, on the contrary, is it true that collective memory comes to an end? Perhaps, these bold claims about the impossibility to forget or the impossibility to remember in a digital era stem from a conception of memory and forgetting that remains confined to the framework of an analog or pre-digital culture. It would be more appropriate to examine the specific ways in which we forget and remember in the current media ecology. Following this line of thought, I would like to briefly discuss two examples of alternative forms of (digital) memory and forgetting explored by Elena Esposito and Serge Bouchardon.

## Re-inventing Forgetting

In ‘Algorithmic Memory and The Right to be Forgotten’ ( 2022), Elena Esposito discusses a judgment that the European Court of Justice issued in 2014 in favor of the plaintiff on case C-131/12 about the ‘right to be forgotten’. The European Court held Google accountable for this ‘excess of memory.’ On the other hand, Google claimed that it could not be held responsible, arguing that the processing of data is carried out by the search engine, and the company does not exercise control over that data. However, according to the European Court, although Google may not be directly responsible for data processing, the search engine’s activity makes that data accessible to users. Esposito remarks that this raises the question of what conception of social memory and forgetting is implied in the ruling:

“Is memory the ability to store information in an archive, even if it is inaccessible? Or does it depend on the ability to find the information when you need it? Is computer memory storage or remembering? Ascribing to Google the management of the right to oblivion implies a clear choice: data are considered forgotten if they are made difficult to find, while social memory should be preserved by the storage of data in the pages of newspapers and in other archives” ( Esposito, 2022, 67–68).

Esposito acknowledges the difficulty of requesting certain information to be erased, among other reasons, because it draws attention to the deleted content, achieving the opposite effect of what is desired. How, then, to deal with forgetting on the web? Esposito suggests that forgetting should follow the logic of algorithmic memory by implementing a procedure that involves multiplying the information instead of erasing it. Therefore, in order to manage forgetting on the web in a way that aligns with algorithmic memory, one could consider implementing a procedure that goes against the conventional practice of deleting or making content unavailable. This involves employing strategies of obfuscation, which generate misleading, false, or ambiguous data alongside each transaction on the web. In practice, this multiplication of information production aims to impede the meaningful contextualization of data ( Esposito, 2022, 73).

This proliferation of information renders each individual piece of data more marginal, getting lost within the vast volume. Rather than being erased, information becomes invisible due to the obfuscation caused by the excess of data.

As Esposito suggests in her conclusion, the issues arising from the digital ecology need to be addressed from a digital perspective, which entails a shift in the reference frame:

“Algorithms participating in communication can implement, for the first time, the classical insight that it might be possible to reinforce forgetting—not by erasing memories but by multiplying them. This requires a radical change in perspective. It does not solve all the problems of digital memory and of the difficulty in controlling the continuous production of an excess of data, but moves these problems to a different and much more effective level: from the reference frame of individuals to that of communication” (76–77).

## Re-inventing memory

In the article ‘Preservation of Digital Literature: From Stored Memory to Reinvented Memory’ ( 2013) Bouchardon and Bachimont address the issue of preservation in the digital age. This is the opposite problem to the one described above. On the one hand, the persistence and availability of digital data raise the issue of the difficulty of forgetting, exemplified by ‘The Right to be Forgotten’. On the other hand, concerning preservation, the digital era is likely the most fragile and complex context in human history. Compared to a medium like books, which have remained virtually unchanged, allowing us to read works produced hundreds or thousands of years ago, the lifespan of digital works is short. They quickly become obsolete. This is explained by the fact that in the digital medium, content is situated at two different levels.

Bachimont makes a distinction between the "inscription form" and the "restitution form" ( Bachimont, 2007). In the context of printed material, both forms are the same, represented by the printed text. However, in the case of digital media, these two forms are separate due to the intervention of computational processes that mediate between them.

The existence of two forms raises the question of how we define content. Is the content what is stored on the hard drive (the resource), or is it the content that appears on the screen (the rendering)? If we preserve the resource but not the representation, can we still speak of preservation? This problem is evident in the field of digital literature, the topic that Bouchardon and Bachimon address in their article, since a digital literary work is not an object —like a printed book— but fundamentally a process that can only exist through actualization. Bouchardon and Bachimon distinguish four possible strategies for the preservation of digital literature: museology, migration, emulation and description.

The museological approach consists in “preserving contents as they are as well as the tools permitting playability. This way, it is not only the information which is preserved, but the technological environment characteristic of a certain time and content” ( Bouchardon & Bachimont, 2013, 187). Migration involves “updating the technical format of the contents so that they should remain compatible with and adapted to the reading tools available in the current technological environment” (188). In emulation “the contents are not made to evolve. Instead, the reading tools of the old formats are simulated on current environments” (188). Lastly, description, an approach that is “counter-intuitive but the most potent on a theoretical level” consists in “discarding recorded contents in as far as they are incomplete, partial or ill-defined. Therefore, it is better to preserve a description of the content which permits us to reproduce it. The description may concern the reproduction of key elements, of the authors’ intention, and the variable media approach, of the graphic appearance, etc;” ( Bouchardon & Bachimont, 2013, 189–90).

The authors compare the description-based preservation method with the preservation model of classical music. How do we know how to play Baroque music today when we do not have recordings of how it was performed in its time? Thanks to the combination of three elements: score, instrument, and instrumental practice. The score serves as a set of instructions for producing music on an instrument. Organology ensures the preservation of instruments and the techniques involved in their creation. Furthermore, through the practice of music, which involves reading scores and playing instruments, knowledge is continuously taught and passed down. Preservation goes hand in hand with constant usage. In this sense, the concept of music serves as a model for preserving a content that cannot be directly recorded. Instead, it relies on saving resources (the score), a player (the instrument), and a practice (music education). This unique musical solution allows for the preservation of a non-variable description of the performance, even in the absence of the original content.

As Bouchardon and Bachimon point out, this preservation model —which does not preserve the content intact but rather the elements necessary for its reconstruction— is essentially an interpretative act. In this model, preservation is synonymous with (re)invention: “Preserving is keeping intact the interpretability of the work to be able to reinvent it. In other words, preserving is saving the knowledge of its re-invention” ( Bouchardon & Bachimont, 2013, 191).

This model, which the authors argue is "from an anthropological point of view" "more valuable and more authentic than the model of printed media which is a memory of storage" (200) effectively embodies one of the central characteristics of collective memory. Like the ontological dynamism of archives, the dialectic between memory and forgetting in the digital age brings us closer to the functioning of collective memory understood as the continuous evolution, change, and adaptation of representations of the past in the present. What collective memory preserves is not an ‘intact’ past. It does not faithfully preserve the facts, characters, or significant events of that past for a community —which does not imply that the representations of the past it preserves are false. Collective memory re-appropriates, adapts, and re-actualizes the facts of the past, along with their affects and values. Re-invention makes room for the creation of new relationships with the past, that is ultimately, the goal of collective memory.

## Conclusion

The digital revolution has had a significant impact on collective memory, resulting in notable changes. Archives, which were once static repositories, have undergone a transformation and now function as dynamic entities. This shift challenges traditional storage methods and promotes new archiving practices that aim to make information more accessible to everyone.

The digital era has prompted a reversal in the relationship between memory and forgetting. What used to be a demanding endeavor requiring significant resources, as memory was a scarce commodity, now appears as an activity we do not even have to worry about. Our visits, purchases, the number of pages we have read in a book, the photos we take on vacation —everything is automatically recorded and preserved. At the same time, the networked operation of our devices can potentially make this data available beyond our intentions and into an unpredictable future.

Now, forgetting is what appears to be a valuable asset to preserve, something that attempts to be regulated through laws like the ‘Right to be Forgotten’. As Makhortykh (2021) suggests, it is interesting to consider whether the ‘Right to be Forgotten’ could also apply collectively and what the consequences would be. What happens if a perpetrator or a victim requests the deletion of information related to their involvement in atrocities? What would occur if a state implicated in crimes against humanity claimed this right?

The flip side of this process is that, despite the apparent persistence of data on the web, the internet is not an ideal mnemonic medium. On the contrary, the fragility of data, caused by factors such as the rapid obsolescence of storage devices, led early critics of the internet in the mid-90s to warn that we might be living in a ‘dark age’ where records will not be preserved. Various responses have been proposed to address the difficulty of preserving data in the digital age, or more specifically, digitally-born content, where content is not an object but fundamentally a process that can only exists through actualization, with interactivity being an essential part of it.

Among these responses, the most interesting one is the intertwining of preservation with reinvention. The metaphor of the ‘Sappho syndrome’ ^5^ is valid in envisioning a memory appropriate to the digital era. If works disappear to the extent that all that remains are fragments and descriptions made by others, comments on comments, all that is left is to reinvent them. But isn't that what collective memory has always done? Reinventing the past based on its remnants and the narratives others have constructed about it.

Digital media has introduced powerful tools that allow us to observe interactions between actors —both human and non-human— with unprecedented precision and abundance of data. What are the consequences of this for the way we conceive of the social? How can a mode of conceiving the social enable us to capture social memory more faithfully? Tarde's proposal, which considers the articulation between the individual and the collective, the micro and the macro, without favoring one at the expense of the other, can provide us with an appropriate conception of the social for thinking about it in the digital age, as well as refining the shortcomings inherited from Halbwachs.

In sum, the extent of the changes introduced by the digital turn is undeniable. However, does it make sense to categorize digital memory as a completely new and radically different form of memory? Does the digital render the concept of collective memory obsolete? As I hope to have showed, collective memory, conceived as a process that is mediated and remediated by multiple media, with the participation of dynamic communities that perform rather than represent the past, is still valid. Furthermore, not only is it valid, but the digital realm materializes its processual nature —which is inscribed in the very archive— brings to the forefront the role of technological mediation and make visible the associations that produce ‘the collective’ without representing collective memory as a disembodied entity that transcend the individuals who comprise it.

## Data Availability

No data associated with this article.
