# Supplementary material for: Memory in the digital age
Source: Open Res Eur. 2024 Jan 12;3:123. Originally published 2023 Aug 4. [Version 2] doi: 10.12688/openreseurope.16228.2 (PMC10509603; doi:10.12688/openreseurope.16228.2)
Supplement: Peer review history

# Reviewer report: Reviewer response for version 1

**DOI:** 10.21956/openreseurope.17518.r34277

**Published:** 2023-09-19

**Authors:** Jessica Ortner

**Affiliations:** 1 Department of English, Germanic and Romance Studies, University of Copenhagen, Copenhagen, Capital Region of Denmark, Denmark

This article is an important intervention into the topic of the digital turn. It is an extremely rich paper. It is well-grounded theoretically and addresses the digital turn in an original and interesting manner.

 The author opposes Hosking’s idea that digital memory is a new form of memory that represents the end of collective memory. Whereas the idea of a digital “turn” of memory studies implies the idea of a revolution, the author claims that the ontology of memory has not changed due to the digitalization of the archive. Instead of changing the idea of memory per se, the digital memory materializes collective memory’s mobile, dynamic, and procedural nature.

 The author criticizes the opinion that the digital medium isn’t understood as a memory medium in its own right (a channel) due to the opinion that the digital memory merely remediates other media. (television/ newspaper/ Cinema). This strong focus on remediation is the reason why the digital memory is understood as a medium that creates an entirely new mnemonic culture.

  

- This argument is interesting but could be put more clearly. After having made a distinction between medium as a communication channel and medium as social practice, it is not stated definitively which kind of media the author wants to advocate. Should the digital memory be defined as a medium in the sense of a channel? And should the notion that the digital memory merely is remediation be opposed or nuanced? Moreover, according to Erll, all mediation is remediation. Is the digital medium understood as another form of remediation? And what about the digitally born media such as online archives? Does their existence mean that the internet should be seen as memory medium in its own right?
- The richness of the paper means that the single parts of the article sometimes seem a bit disjointed. I would recommend some more methodological signposts to chatch up on the overall argument.

**The social**

 Even though the ontology of memory has not changed, the author suggests that the idea of collective memory needs to be rethought. Not memory, the argument goes, but the idea of the social as it was developed by Halbwachs is no longer adequate. Instead, she suggests that Tarde’s idea of the social which is more useful.

 According to Tarde, social relationships not only occur between human beings but in principal, every element has the capability to associate. Every entity is part of a complex network of social relations, but this collective cannot be subsumed under a structure because every entity always can interconnect with new entities.

 Even though “the individual does not exist” according to Tarde, the author suggests that the individual in this model is freed from homogeneous structures. Transferred to the digital area, this means that the screen allows people to switch forth and back between individuality and being part of some kind of aggregate (social association). Thus, all interconnections are provisory and instable.

 One could almost say that heterogeneity gets law.  

- There seems to be a slippage when the article switches from the theoretical description of the social according to Tarde to her usage of this theory on the digital network. Especially the connection between Tarde’s monads and the digital is a bit unclear. What exactly is the digital counterpart to the monads? Furthermore, whereas heterogenicity and freedom from structures seems to be predominant in Tarde’s idea of the social, algorithms - according to the author - limit that very freedom.
- It is an interesting and important recognition that algorithms have an impact on collective memory and that we should be aware of its power. Perhaps that could also be presented as one main argument in the introduction and combine it with the later argument that “forgetting should follow the logic of algorithmic memory.” In short, it could be made clear that the article will show that both memory and forgetting in the digital age have an impact on collective memory. After all, the social is only one of the basic preconditions of collective memory that are negotiated

(Just a thought: One could also argue that if the social is performative, people could choose to reinforce the same aggregate again and again and thereby actively attempt to shape a stable social frame. I agree that the homogeneous entity of the social does not exist in itself, but it certainly is performatively created.)

**Archive**

 The record about the new conditions of the archive regarding archived objects, the place and the practice of archiving are interesting and informative. However, I miss that the author choses sites – e.g. regarding the question of the placelessness of the archive versus the argument of the “place-making.” I find the new role and the opposition against Assmanns idea of the archive convincing. (a thought: Perhaps, the author could consider linking the argument about the creativity of the archive to Hal Foster’s idea of the archive)

- When the author begins with the section about the archive, it could be useful to remind the reader *why* we now are switching to the topic of the archive. picking up the basic hypothesis. As I get it, the argument is that even though memory has not a changed ontologically, the archive has. As the author puts it “The archive, conceived as the guarantor of the stability of collective memory, undergoes an ontological change characterized by dynamism and mobility.” 
- I find the part about the role of the algorithm in the digital archive especially fascinating. Again, it could be useful to point back to the argument that the algorithm influences cultural memory, which the author made in the former section.

Here, I actually think that Halbwachs model of the social frameworks could be used in order to demonstrate the revolutionary aspect that the democratized or automatized selection process now determines what should be preserved and what can be forgotten. That is, these processes take over the task of drawing a demarcation line between forgettable and memorable pasts by making it arbitrary and unstable.

**Human rights**

- Also the section about human right could be introduced with some metatext. Perhaps the assumption that “the link between memory and human rights explains the positive role assigned to the act of remembering and the corresponding stigmatization of forgetting” should be modified a bit. It is certainly one, but not the only explanation for the focus on remembering.
- The linkage between the digital memory and the new trend to focus on forgetting is highly interesting. However, it is introduced rather late and comes as a surprise, since the article starts with debating memory and the social. Also, one could add that Assmann has pointed out that forgetting is norm and remembering the exception.
- Finally, also the second argument about the fragility of the digital era is interesting. Here one could perhaps add a consideration what this means for the general idea of cultural memory that it is a long-term memory of the community.

I enjoyed reading this paper and believe that it will be an important contribution to the field

Is the argument persuasive and supported by appropriate evidence?

Yes

Is the work clearly and cogently presented?

Partly

Is the topic of the essay discussed accurately in the context of the current literature?

Yes

Does the essay contribute to the cultural, historical, social understanding of the field?

Yes

Reviewer Expertise:

My field of expertise is literary studies and memory studies. I have not worked extensively with the topic of the digital turn but think that my knowledge about the methodology of memory studies enables me to assess this paper

I confirm that I have read this submission and believe that I have an appropriate level of expertise to confirm that it is of an acceptable scientific standard, however I have significant reservations, as outlined above.

# Reviewer report: Reviewer response for version 1

**DOI:** 10.21956/openreseurope.17518.r34274

**Published:** 2023-09-15

**Authors:** Eivind Røssaak

**Affiliations:** 1 Department of Scholarship and Collections, National Library of Norway, Oslo, Norway

The article “Memory in the digital age” is rich and well argued. It deals with the status of collective memory in a world changed and challenged by digital technologies. The article gives an apt overview of the classic definitions of collective memory before it goes on to discuss the impact of digital technologies. It refers to relevant literature on how the use of digital technologies have drastically altered contemporary memory, to researchers like Andrew Hoskins, who discusses how the saturation of digital technologies may imply the “end of collective memory”. The author disagrees and builds a different argument. The author’s argument has a very interesting twist. To a large extent the author seems to agree with many of the critical contributions to the field who argue that “digital memory” is a game changer. However, the author explains how digital memory does not necessarily undermine the use of the term “collective memory”, rather the opposite, digital memory shows us even better what collective memory has always been. The author writes: “Digital memory is not a new form of memory and, far from representing the end of collective memory, it materializes and puts into practice the characteristics with which we have defined collective memory since the inception of the field. In fact, we could argue that what we have always advocated about collective memory finds its true realization in the digital era” (p.4).

 After discussing the early formation of collective memory studies, the author goes on to discuss the nineteenth-century social theorist Gabriel Tarde, who Bruno Latour has called a digital theorist 100 years ahead of his time. Tarde may provide a fresh view on how to address memory after the digital. Rather that analyzing norms and structures as Durkheim did, Tarde promoted the idea that the social behaviour of all phenomena in the universe contributed to what constitutes a social fact. However, Tarde needed better tools to trace the relations and associations of multiple events in society. Via Latour’s suggestion, the author argues that digital technologies may be precisely such a tool; it can “make visible the associations that produce ‘the collective’” (p. 6). Digital technologies show us how memories are dynamic, evolving, and fluid, and Tarde’s theory “allows us to explore collective memory as a process encompassing both (human) connectedness and (automated) connectivity” (p. 7). These technologies are like “archives in motion”, much closer to living memory than to a static archive constituting a fixed place, object, and practice. Rather than seeing digital technologies as an end to memory, as I would suggest Pierre Nora proposes ( Røssaak 2018), the author suggests in a convincing way that several aspects of the use of digital technologies in society answers to the ways collective memory is made. Digital memory is in other words not a radically different form of memory but can alert us to the dynamisms of memory that have always been there.

 I may not agree with the author’s sense of optimism, when it is stated that “[d]igital media has introduced powerful tools that allow us to observe interactions between actors […] with unprecedented precision and abundance of data” (p. 11). Relying too much on the quantitative approaches of digital methods may lead to a naïve techno-positivism, and furthermore, relying on the continuous availability of an “abundance of data” may be detrimental: On the one hand, big data may promote new regimes of surveillance and manipulation, and on the other, digital repositories and communication platforms may suddenly disappear, due to a crash or business considerations such as in the case with *GeoCities *which suddenly disappeared in 2009, and who knows what will happen to *Twitter* (now, *X*) as Elon Musk changes its protocols? Nevertheless, the article invites the reader to ponder several other questions, as well. If digital technologies materialize the processes of collective memory, how could we explore pre-digital memory formations with a view to these materializations? Do digital memory-processes teach us something about pre-digital memory-processes?

 A few details: The author asserts on page 3 that the “digital turn” has “revolutionized” culture “on a global scale”. This assertion may be in conflict with the research question further down on the same page: “Is it an evolution or a revolution?” A less dramatic term than “revolutionized” may be adequate. Furthermore, from the article’s context it makes sense to state that “memory is always mediated” (p. 3 and 4), but I think it is always more precise to say that “collective” or “social” memory is always mediated, because “memory” generally speaking, as we all know, refers to many things, i.e. biological or sensory memory is a different story, but is nevertheless, relevant when, say, Bergson is mentioned as part of the picture.

Is the argument persuasive and supported by appropriate evidence?

Yes

Is the work clearly and cogently presented?

Yes

Is the topic of the essay discussed accurately in the context of the current literature?

Yes

Does the essay contribute to the cultural, historical, social understanding of the field?

Yes

Reviewer Expertise:

Media history, archival history, library history, cultural theory, archival art

I confirm that I have read this submission and believe that I have an appropriate level of expertise to confirm that it is of an acceptable scientific standard.

# Reviewer report: Reviewer response for version 1

**DOI:** 10.21956/openreseurope.17518.r34497

**Published:** 2023-09-04

**Authors:** Manuel Menke

**Affiliations:** 1 University of Copenhagen, Copenhagen, Denmark

The essay at hand challenges recent contributions in memory studies, which – as the author states – implicitly or explicitly suggest that the digital turn changed memory in such fundamental ways that it can no longer be captured by the concept of collective memory. The core argument is developed that instead of dismissing collective memory as a framework to describe memory in the digital age, it is, in fact, digital memory, which eventually “materializes and puts into practice the characteristics with which we have defined collective memory since the inception of the field (p.4).” The manuscript starts with this critical main hypothesis and then makes the case for collective memory as a continuously useful concept by rethinking the social under the conditions of the digital turn. The reader is also introduced to several sections diving into more specific changes, such as the altered function and constitution of the archive and the reconfiguration of the relationship between memory and forgetting.

 Overall, the argumentation is well-developed and presented in a comprehensive and convincing manner. The manuscript is of high quality and theoretical rigor, meaning the chapters are rich and do justice to the complexity of the matter. Important literature is not only covered but also in most cases critically discussed and well-integrated. The essay thereby will be a valuable and thought-provoking contribution.

 I would like to make two suggestions for a revision to strengthen the essay and the case it tries to make, which also aims at anticipating and countering potential critique:

**1) **The major hypothesis is presented in a rather provocative way in the abstract, plain language summary, and introduction by pointing at the conceptual shortcomings in the work of others. While there is nothing wrong with that and we should embrace challenging each other’s work, I would argue that an author should do everything in their power to present a strong case if strong claims are made. In this essay, however, the criticism is not supported by enough evidence and lacks engagement in the work that is being criticized. The only paragraph on that matter is:

 “In the recent debate, the prevailing idea is that digital memory represents a radical change, giving rise to a new form of memory referred to as “algorithmic memory,” “connective memory,” “new memory,” or “the memory of the multitude” (Blom, 2017; Garde-Hansen et al., 2009; Makhortykh, 2021; Hoskins, 2009; Hoskins, 2011; Hoskins, 2018b). According to these perspectives, digital memory not only implies a different ontology but, in more radical proposals, even entails the “end of collective memory” (Hoskins, 2018a).”

 I would argue that this is not enough to draw from and an engagement in the authors’ line of argumentation is needed; and not only with their labeling or final claim. As of yet, it is not demonstrated how these authors actually introduce an ontological difference; and if so, how they argue for it. The essay would benefit from a chapter making that case. Just to play devil’s advocate: If I were to “defend” these authors, I would argue that they probably thought of new modes of digital memory making, memory work, mnemonic infrastructures, etc. instead of claiming that these replace collective memory as a concept that describes the social dimension and constitution of memory. Even in the chapter by Hoskins in which he provocatively asks if the multitude of memory means the end of collective memory, he advocates for an “updating” or “re-thinking” and does not explicitly promote a complete dismissal of decades of discourse about collective memory. And yet, he also writes: “I now turn to examine the rise of the multitude and the memory of the multitude as a challenge to previous formations of memory imagined and presumed as collective, but also as a solution to the re-thinking of individual and social relations now blurred through their immersion in digital networks and archives.” Here it would be important to discuss in the essay why his and other approaches might mislead us with their new concepts to then make the case why “collective memory” might be a superior path forward. I agree that there is a lot to criticize, such as the trend to arbitrarily put new terms in front of “memory” obfuscating (established) meaning or that the argumentation of Hoskins in his chapter paints the picture of dystopian digital memory and only with this (maybe false or extreme) premise announces “the end of collective memory”. In my opinion, there is a lot to discuss but it needs to be covered substantially in the essay.

**2) **My second suggestion is to work more on the chapter “Rethinking the social” and in particular on the integration of Tarde’s conception of the social. I am not familiar with his work so I speak for a group of readers coming to this essay without prior knowledge. I was not able to follow and understand the theoretical explanation to a degree to which I could see the benefit and how it relates to memory and the digital. I assume that summarizing the core building blocks of this theory is extremely difficult and for academics who locate the social between human beings, in social contexts, and by the means of culture and cultural artifacts, etc. it is hard to fathom how “social behavior is inherent in all phenomena in the universe” and what a “monad” even is. It is not my intention to dismiss this theory and others might have better access to it, yet, I would argue that the chapter needs more examples and explanations of how this theory plays out in the digital and in collective memory. It remains too abstract and expects too much knowledge from the readers to make these connections for themselves. Since this is the foundation of the argument for why the digital enables the social and thereby collective memory, it should be as accessible and clear as possible.

 I would like to stress that all other parts of the essay do a great job of outlining concepts, changes, and their impact. It is well-written and an overall inspiring read.

**Minor remarks:**

- Several times, it is stated that all memory is mediated. This should be explained because I am not sure where memory shared in co-presence (in a conversation) would fall if that was the case. At least in my understanding as a media scholar, media does not include the body. So a version of “almost all memory is mediated” may make sense.
- p. 3 bottom left column: “…[is it] better to think the digital as a remediation of the older media?”
- Footnote 2: “Tar[r]de”
- p. 5 bottom left column: “…that constitute the group but [do] not need to be shared…”

Thank you for the opportunity to read and review this essay. I hope my remarks are helpful and I was able to formulate my criticism in a constructive way. All the best for the revision and I am looking forward to the next version. It is already an impressive piece.

Is the argument persuasive and supported by appropriate evidence?

Partly

Is the work clearly and cogently presented?

Partly

Is the topic of the essay discussed accurately in the context of the current literature?

Yes

Does the essay contribute to the cultural, historical, social understanding of the field?

Yes

Reviewer Expertise:

Memory/nostalgia; media studies; digital publics; social media communication

I confirm that I have read this submission and believe that I have an appropriate level of expertise to confirm that it is of an acceptable scientific standard, however I have significant reservations, as outlined above.
